# Supplementary material for: The Epigenetic Modifiers HDAC2 and HDAC7 Inversely Associate with Cancer Stemness and Immunity in Solid Tumors
Source: Int J Mol Sci. 2024 Jul 17;25(14):7841. doi: 10.3390/ijms25147841 (PMC11277355; doi:10.3390/ijms25147841)
Supplement: Supplementary file 1 [file ijms-25-07841-s001.zip › ijms-3056444-supplementary.pdf]

The Epigenetic Modifiers HDAC2 and HDAC7 Inversely Associate with Cancer Stemness and Immunity in Solid Tumors

Kacper Maciejewski [1], Marek Giers [1], Urszula Oleksiewicz [2,3,*] and Patrycja Czerwinska [1,2,3,*]

[1] Undergraduate Research Group "Biobase", Poznan University of Medical Sciences, 61-701 Poznan, Poland; maciejewskikacper@mensa.org.pl (K.M.); marek.giers00@gmail.com (M.G.)
[2] Department of Cancer Immunology, Poznan University of Medical Sciences, 61-866 Poznan, Poland
[3] Department of Diagnostics and Cancer Immunology, Greater Poland Cancer Centre, 61-866 Poznan, Poland
* Correspondence: u.oleksiewicz@gmail.com (U.O.); czerwinska.patrycja@ump.edu.pl (P.C)

Table of content

Figure S1 2
Figure S2 3
Figure S3 4
Table S1 5
Figure S4 19
Figure S5 20
Figure S6 21
Figure S7 21
Figure S8 22
Figure S9 23
Figure S10 24
Figure S11 25
Figure S12 26
Figure S13 27
Figure S14 28
Figure S15 29
Figure S16 30

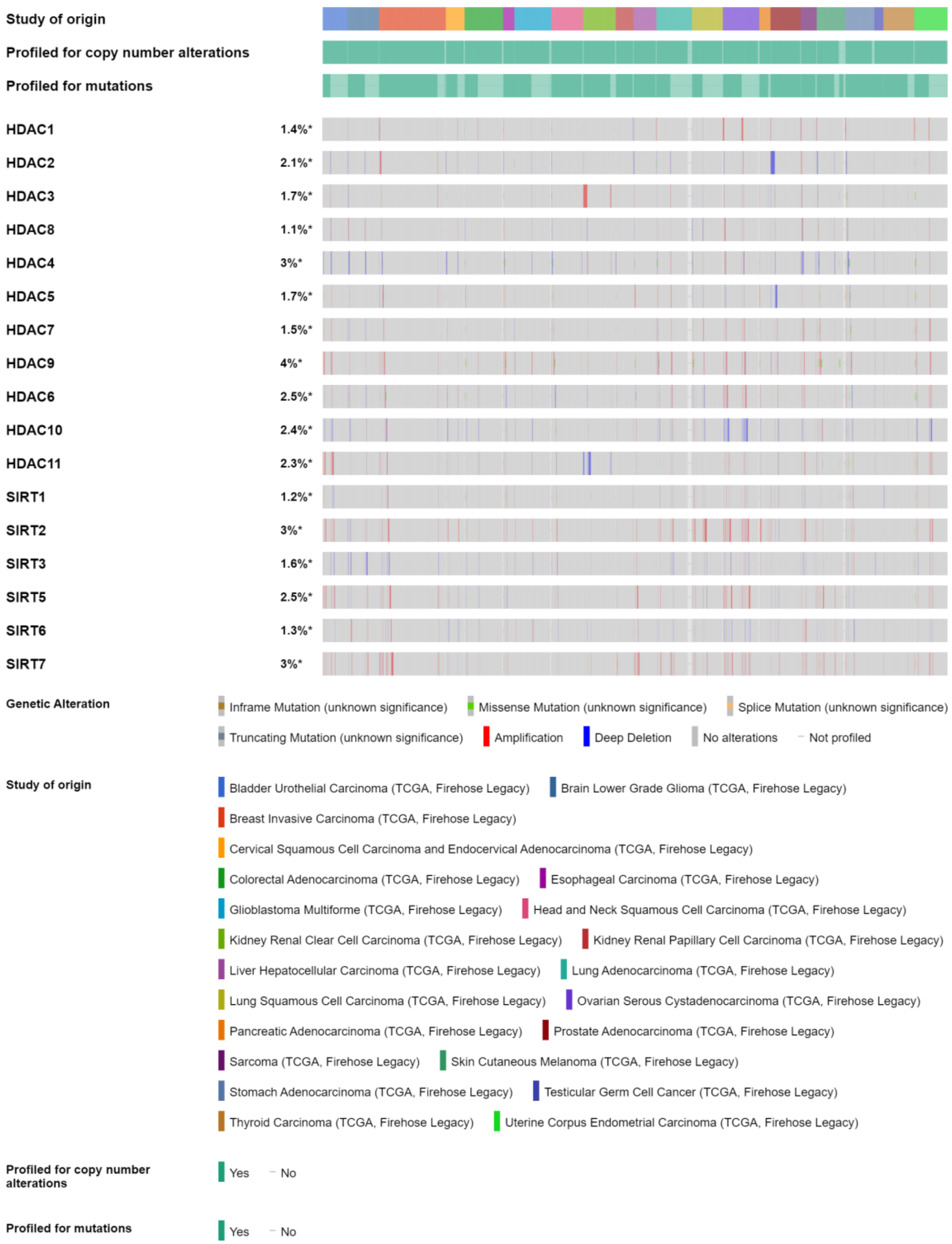

Figure S1. The alteration frequencies in HDAC family members. Distinct patterns of mutations are observed in HDAC members across solid tumors. The color-coded TCGA studies and the types of genetic alterations (inframe, missense, splice, truncating mutation, amplification, or deep deletion) are described in the legend. The figure is taken directly from the cBioportal database (combined virtual study, https://bit.ly/4a3NQh7, accessed on 10 March 2024).

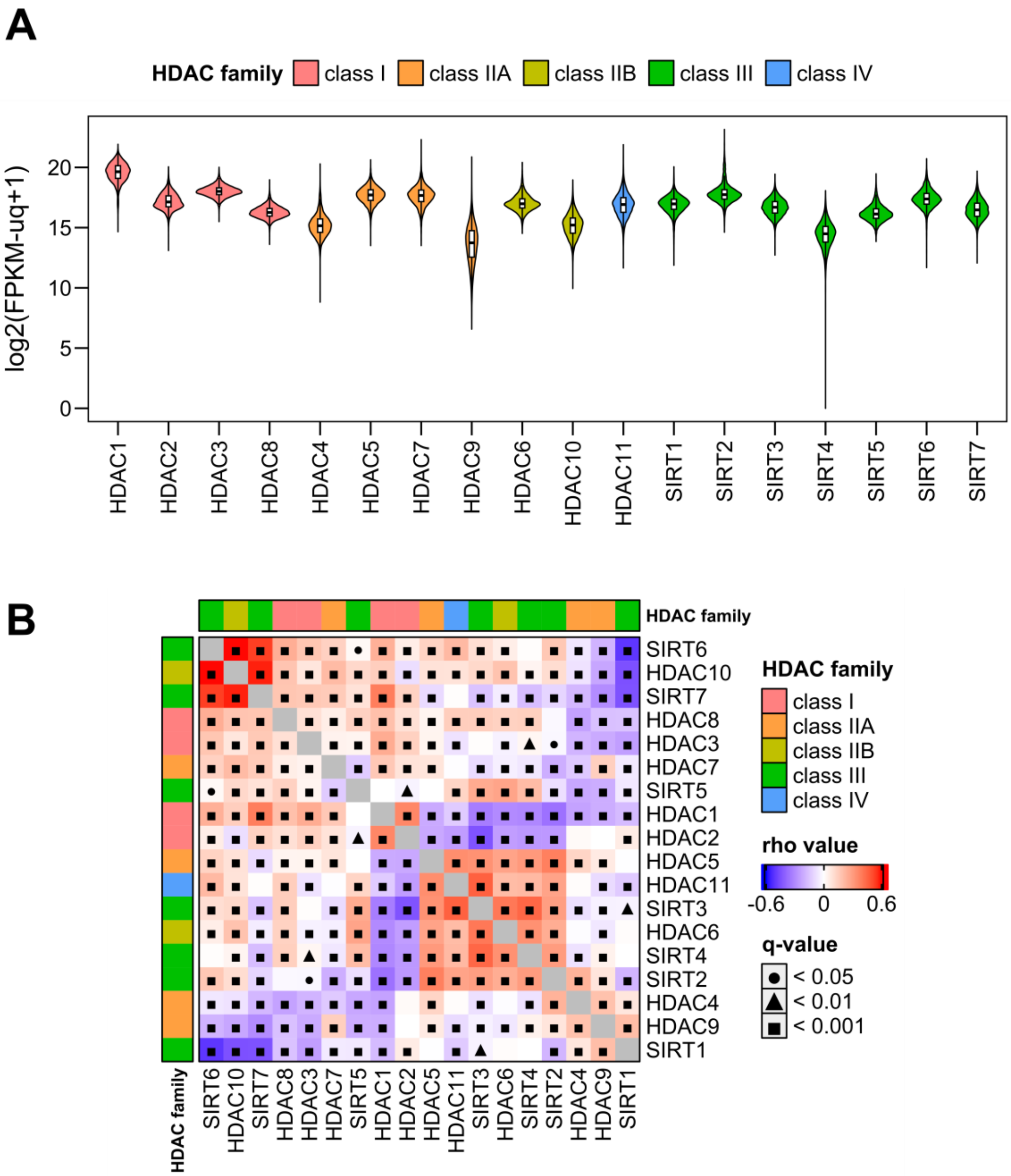

Figure S2. HDAC family gene expression in the GDC PANCAN dataset (including only TCGA studies of solid tumors with more than 100 RNA-seqV2 available samples). (A) Distribution for each HDAC family gene expression. (B) Co-expression of HDAC family genes. Spearman's test with asymptotic t-test for p-values. Benjamin-Hochberg for multiple testing corrections.

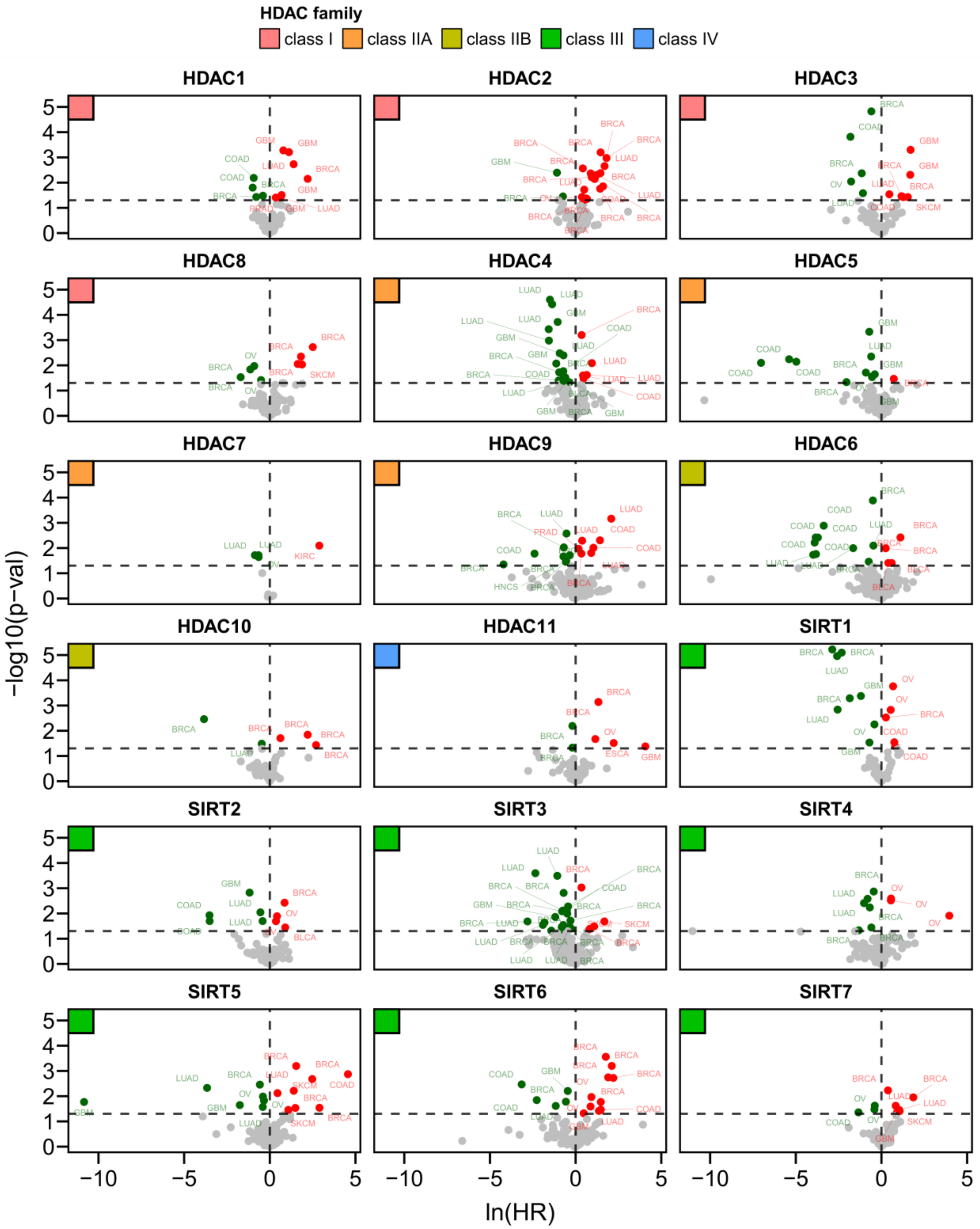

Figure S3. The association of HDAC family members' expression with cancer patients' overall survival using the Prognoscan database. Dot plots demonstrate the ln-normalized hazard ratio of death - either lower (green dots) or higher (red dots) - for cancer patients expressing higher levels of specific HDAC members. Only statistically significant data ($p < 0.05$) are color-coded (for further details see Table 1).

Table S1. Datasets employed for the analysis of associations between HDAC family members and patients' overall survival from the Prognoscan database. Each row in this table is represented by a single dot in Figure S3. *P.* is an abbreviation for prognosis of patients expressing higher levels of specific HDAC members: the arrow up/green color represents better prognosis, and the arrow down/red color represents worse prognosis. Cancer types are color-coded.

HDAC1

CLASS	DATASET	P.	CANCER TYPE	SUBTYPE	COHORT	AUTHOR	ARRAY TYPE	PROBE ID	N	CUTPOINT	COX P-VALUE	ln(HR)	HR [95% CI low - upp]
I	GSE7378	↓	Breast cancer		UCSF	Zhou	U133AAofAv2	201209_at	54	0.78	0.007083	**2.21**	9.08 [1.82 - 45.26]
	GSE31210		Lung cancer	Adenocarcinoma	NCCRI	Okayama	HG-U133_Plus_2	201209_at	204	0.53	0.001863	**1.39**	4.02 [1.67 - 9.67]
	GSE4271-GPL96		Brain cancer	Astrocytoma	MDA	Phillips	HG-U133A	201209_at	77	0.52	0.000620	**1.12**	3.06 [1.61 - 5.80]
	MGH-glioma		Brain cancer	Glioma	CBTTB, MGH, BWH, CH	Nutt	HG-U95A	38771_at	50	0.68	0.000525	**0.79**	2.19 [1.41 - 3.42]
	GSE4412-GPL96		Brain cancer	Glioma	UCLA (1996-2003)	Freije	HG-U133A	201209_at	74	0.16	0.030980	**0.69**	1.99 [1.07 - 3.73]
	GSE13213		Lung cancer	Adenocarcinoma	Nagoya (1995-1999, 2002-2004)	Tomida	G4112F	A_23_P114656	117	0.82	0.039020	**0.62**	1.85 [1.03 - 3.32]
	MGH-glioma		Brain cancer	Glioma	CBTTB, MGH, BWH, CH	Nutt	HG-U95A	476_s_at	50	0.48	0.040029	**0.57**	1.77 [1.03 - 3.05]
	GSE16560		Prostate cancer		Sweden (1977-1999)	Sboner	6K DASL	DAP4_1042	281	0.42	0.039140	**0.35**	1.42 [1.02 - 1.97]
	GSE12276	↑	Breast cancer		EMC	Bos	HG-U133_Plus_2	201209_at	204	0.44	0.032950	**-0.40**	0.67 [0.47 - 0.97]
	GSE1379		Breast cancer		MGH (1987-2000)	Ma	Arcturus 22k	12350	60	0.38	0.037120	**-0.79**	0.45 [0.21 - 0.95]
	GSE14333		Colorectal cancer		Melbourne	Jorissen	HG-U133_Plus_2	201209_at	226	0.12	0.006498	**-0.94**	0.39 [0.20 - 0.77]
	GSE17536		Colorectal cancer		MCC	Smith	HG-U133_Plus_2	201209_at	145	0.43	0.015742	**-1.00**	0.37 [0.16 - 0.83]

HDAC2

CLASS	DATASET	P.	CANCER TYPE	SUBTYPE	COHORT	AUTHOR	ARRAY TYPE	PROBE ID	N	CUTPOINT	COX P-VALUE	ln(HR)	HR [95% CI low - upp]
	GSE6532-GPL570		Breast cancer		GUYT	Loi	HG-U133_Plus_2	242141_at	87	0.63	0.001057	**1.80**	6.04 [2.06 - 17.73]

	GSE6532-GPL570		Breast cancer		GUYT	Loi	HG-U133_Plus_2	242141_at	87	0.63	0.001057	**1.80**	6.04 [2.06 - 17.73]
	GSE31210		Lung cancer	Adenocarcinoma	NCCRI	Okayama	HG-U133_Plus_2	201833_at	204	0.89	0.002214	**1.69**	5.44 [1.84 - 16.08]
	GSE17537		Colorectal cancer		VMC	Smith	HG-U133_Plus_2	201833_at	55	0.18	0.014067	**1.60**	4.93 [1.38 - 17.64]
	GSE1456-GPL97		Breast cancer		Stockholm (1994-1996)	Pawitan	HG-U133B	242141_at	159	0.74	0.000632	**1.46**	4.32 [1.87 - 9.99]
	GSE1456-GPL97		Breast cancer		Stockholm (1994-1996)	Pawitan	HG-U133B	242141_at	159	0.74	0.004200	**1.45**	4.26 [1.58 - 11.51]
	GSE12093		Breast cancer		IO, NCI, TUM, CCF (1992-2000)	Zhang	HG-U133A	201833_at	136	0.89	0.017386	**1.43**	4.17 [1.29 - 13.54]
	GSE31210		Lung cancer	Adenocarcinoma	NCCRI	Okayama	HG-U133_Plus_2	201833_at	204	0.87	0.005002	**1.22**	3.38 [1.44 - 7.93]
	GSE3494-GPL97		Breast cancer		Uppsala (1987-1989)	Miller	HG-U133B	242141_at	236	0.45	0.007181	**1.11**	3.04 [1.35 - 6.85]
	GSE11121	↓	Breast cancer		Mainz (1988-1998)	Schmidt	HG-U133A	201833_at	200	0.88	0.006092	**0.94**	2.56 [1.31 - 5.00]
**I**	GSE3494-GPL96		Breast cancer		Uppsala (1987-1989)	Miller	HG-U133A	201833_at	236	0.73	0.004321	**0.88**	2.41 [1.32 - 4.42]
	GSE4922-GPL97		Breast cancer		Uppsala (1987-1989)	Ivshina	HG-U133B	242141_at	249	0.73	0.044340	**0.66**	1.94 [1.02 - 3.71]
	GSE1378		Breast cancer		MGH (1987-2000)	Ma	Arcturus 22k	5273	60	0.78	0.042382	**0.62**	1.87 [1.02 - 3.42]
	GSE13213		Lung cancer	Adenocarcinoma	Nagoya (1995-1999, 2002-2004)	Tomida	G4112F	A_23_P122304	117	0.88	0.018993	**0.50**	1.64 [1.08 - 2.49]
	GSE4922-GPL96		Breast cancer		Uppsala (1987-1989)	Ivshina	HG-U133A	201833_at	249	0.69	0.048328	**0.49**	1.63 [1.00 - 2.66]
	GSE12276		Breast cancer		EMC	Bos	HG-U133_Plus_2	201833_at	204	0.45	0.002730	**0.42**	1.53 [1.16 - 2.02]
	GSE9891		Ovarian cancer		AOCS, RBH, WH, NKI-AVL (1992-2006)	Tothill	HG-U133_Plus_2	201833_at	278	0.81	0.037140	**0.37**	1.45 [1.02 - 2.07]
	E-TABM-158	↑	Breast cancer		UCSF, CPMC (1989-1997)	Chin	HG-U133A	201833_at	117	0.26	0.035260	**-0.74**	0.48 [0.24 - 0.95]
	GSE4412-GPL96		Brain cancer	Glioma	UCLA (1996-2003)	Freije	HG-U133A	201833_at	74	0.23	0.004040	**-1.09**	0.34 [0.16 - 0.71]

HDAC3

CLASS	DATASET	P.	CANCER TYPE	SUBTYPE	COHORT	AUTHOR	ARRAY TYPE	PROBE ID	N	CUTPOINT	COX P-VALUE	ln(HR)	HR [95% CI low - upp]

	GSE4271-GPL96		Brain cancer	Astrocytoma	MDA	Phillips	HG-U133A	216326_s_at	77	0.57	0.000502	**1.70**	5.45 [2.10 - 14.17]
	GSE4412-GPL96		Brain cancer	Glioma	UCLA (1996-2003)	Freije	HG-U133A	216326_s_at	74	0.15	0.004909	**1.68**	5.35 [1.66 - 17.22]
	GSE19234		Skin cancer	Melanoma	NYU	Bogunovic	HG-U133_Plus_2	216326_s_at	38	0.89	0.037355	**1.54**	4.65 [1.09 - 19.77]
	GSE17536	↓	Colorectal cancer		MCC	Smith	HG-U133_Plus_2	216326_s_at	177	0.58	0.037479	**1.27**	3.55 [1.08 - 11.72]
	E-TABM-158		Breast cancer		UCSF, CPMC (1989-1997)	Chin	HG-U133A	216326_s_at	117	0.51	0.034598	**1.17**	3.24 [1.09 - 9.61]
	E-TABM-158		Breast cancer		UCSF, CPMC (1989-1997)	Chin	HG-U133A	216326_s_at	117	0.51	0.034598	**1.17**	3.24 [1.09 - 9.61]
**I**	GSE13213		Lung cancer	Adenocarcinoma	Nagoya (1995-1999, 2002-2004)	Tomida	G4112F	A_23_P7388	117	0.85	0.028928	**0.46**	1.58 [1.05 - 2.38]
	GSE9893		Breast cancer		Montpellier, Bordeaux, Turin (1989-2001)	Chanrion	MLRG Human 21K V12.0	19404	155	0.23	0.000015	**-0.59**	0.55 [0.43 - 0.72]
	jacob-00182-UM		Lung cancer	Adenocarcinoma	UM	Shedden	HG-U133A	216326_s_at	178	0.22	0.026234	**-1.08**	0.34 [0.13 - 0.88]
	GSE2034	↑	Breast cancer		Rotterdam (1980-1995)	Wang	HG-U133A	216326_s_at	286	0.32	0.004269	**-1.15**	0.32 [0.14 - 0.70]
	GSE8841		Ovarian cancer		Milan (1992-2003)	Marchini	G4100A	12592	81	0.12	0.009045	**-1.77**	0.17 [0.05 - 0.64]
	GSE14333		Colorectal cancer		Melbourne	Jorissen	HG-U133_Plus_2	216326_s_at	226	0.15	0.000153	**-1.81**	0.16 [0.06 - 0.42]

HDAC8

CLASS	DATASET	P.	CANCER TYPE	SUBTYPE	COHORT	AUTHOR	ARRAY TYPE	PROBE ID	N	CUTPOINT	COX P-VALUE	ln(HR)	HR [95% CI low - upp]
	GSE1456-GPL97		Breast cancer		Stockholm (1994-1996)	Pawitan	HG-U133B	223909_s_at	159	0.89	0.001889	**2.51**	12.30 [2.53 - 59.91]
	GSE19234		Skin cancer	Melanoma	NYU	Bogunovic	HG-U133_Plus_2	223909_s_at	38	0.50	0.009270	**1.88**	6.53 [1.59 - 26.84]
**I**	GSE1456-GPL97	↓	Breast cancer		Stockholm (1994-1996)	Pawitan	HG-U133B	223909_s_at	159	0.89	0.004485	**1.82**	6.17 [1.76 - 21.66]
	GSE1456-GPL97		Breast cancer		Stockholm (1994-1996)	Pawitan	HG-U133B	223909_s_at	159	0.89	0.008831	**1.63**	5.11 [1.51 - 17.34]
	GSE9891	↑	Ovarian cancer		AOCS, RBH, WH, NKI-AVL (1992-2006)	Tothill	HG-U133_Plus_2	223909_s_at	278	0.48	0.038437	**-0.51**	0.60 [0.37 - 0.97]

	GSE17260		Ovarian cancer		Niigata (1997-2008)	Yoshihara	G4112A	A_23_P84922	110	0.27	0.010653	**-0.91**	0.40 [0.20 - 0.81]
	GSE1378		Breast cancer		MGH (1987-2000)	Ma	Arcturus 22k	14784	60	0.23	0.014568	**-1.14**	0.32 [0.13 - 0.80]
	GSE9195		Breast cancer		GUYT2	Loi	HG-U133_Plus_2	223345_at	77	0.12	0.029415	**-1.70**	0.18 [0.04 - 0.84]

HDAC4

CLASS	DATASET	P.	CANCER TYPE	SUBTYPE	COHORT	AUTHOR	ARRAY TYPE	PROBE ID	N	CUTPOINT	COX P-VALUE	ln(HR)	HR [95% CI low - upp]
	jacob-00182-CANDF		Lung cancer	Adenocarcinoma	CAN/DF	Shedden	HG-U133A	204225_at	82	0.71	0.008168	**0.95**	2.58 [1.28 - 5.21]
	GSE4573		Lung cancer	Squamous cell carcinoma	Michigan (1991-2002)	Raponi	HG-U133A	204225_at	129	0.85	0.024112	**0.65**	1.91 [1.09 - 3.34]
	GSE17536	↓	Colorectal cancer		MCC	Smith	HG-U133_Plus_2	228813_at	177	0.79	0.033066	**0.46**	1.58 [1.04 - 2.42]
	GSE31210		Lung cancer	Adenocarcinoma	NCCRI	Okayama	HG-U133_Plus_2	1554322_a_at	204	0.86	0.025116	**0.43**	1.53 [1.05 - 2.22]
	GSE9893		Breast cancer		Montpellier, Bordeaux, Turin (1989-2001)	Chanrion	MLRG Human 21K V12.0	4794	155	0.85	0.000630	**0.35**	1.42 [1.16 - 1.73]
	GSE13507		Bladder cancer		CNUH	Kim	Human-6 v2	ILMN_1764396	165	0.38	0.046787	**-0.36**	0.70 [0.49 - 0.99]
	GSE4271-GPL96		Brain cancer	Astrocytoma	MDA	Phillips	HG-U133A	204225_at	77	0.78	0.049587	**-0.57**	0.57 [0.32 - 1.00]
**IIA**	GSE4412-GPL96		Brain cancer	Glioma	UCLA (1996-2003)	Freije	HG-U133A	204225_at	74	0.53	0.030301	**-0.60**	0.55 [0.32 - 0.94]
	GSE6532-GPL570		Breast cancer		GUYT	Loi	HG-U133_Plus_2	228813_at	87	0.28	0.028295	**-0.68**	0.50 [0.27 - 0.93]
	GSE6532-GPL570		Breast cancer		GUYT	Loi	HG-U133_Plus_2	228813_at	87	0.28	0.028295	**-0.68**	0.50 [0.27 - 0.93]
	GSE3141	↑	Lung cancer	NSCLC	Duke	Bild	HG-U133_Plus_2	228813_at	111	0.71	0.041136	**-0.70**	0.50 [0.25 - 0.97]
	GSE3141		Lung cancer	NSCLC	Duke	Bild	HG-U133_Plus_2	204225_at	111	0.11	0.004024	**-0.71**	0.49 [0.30 - 0.80]
	GSE17536		Colorectal cancer		MCC	Smith	HG-U133_Plus_2	204225_at	145	0.26	0.017001	**-0.72**	0.49 [0.27 - 0.88]
	GSE14333		Colorectal cancer		Melbourne	Jorissen	HG-U133_Plus_2	204225_at	226	0.23	0.021871	**-0.74**	0.48 [0.26 - 0.90]
	GSE4412-GPL97		Brain cancer	Glioma	UCLA (1996-2003)	Freije	HG-U133B	228813_at	74	0.45	0.003311	**-0.93**	0.40 [0.21 - 0.73]

	GSE1456-GPL96		Breast cancer		Stockholm (1994-1996)	Pawitan	HG-U133A	204225_at	159	0.39	0.018982	**-0.96**	0.38 [0.17 - 0.85]
	GSE9195		Breast cancer		GUYT2	Loi	HG-U133_Plus_2	204225_at	77	0.52	0.040023	**-0.99**	0.37 [0.14 - 0.96]
	GSE4271-GPL97		Brain cancer	Astrocytoma	MDA	Phillips	HG-U133B	228813_at	77	0.65	0.000191	**-1.05**	0.35 [0.20 - 0.61]
	MGH-glioma		Brain cancer	Glioma	CBTTB, MGH, BWH, CH	Nutt	HG-U95A	38271_at	50	0.70	0.008456	**-1.13**	0.32 [0.14 - 0.75]
	GSE31210		Lung cancer	Adenocarcinoma	NCCRI	Okayama	HG-U133_Plus_2	228813_at	204	0.64	0.000038	**-1.37**	0.26 [0.13 - 0.49]
	GSE31210		Lung cancer	Adenocarcinoma	NCCRI	Okayama	HG-U133_Plus_2	204225_at	204	0.53	0.000025	**-1.50**	0.22 [0.11 - 0.45]
	GSE31210		Lung cancer	Adenocarcinoma	NCCRI	Okayama	HG-U133_Plus_2	204225_at	204	0.52	0.001046	**-1.56**	0.21 [0.08 - 0.53]
	GSE31210		Lung cancer	Adenocarcinoma	NCCRI	Okayama	HG-U133_Plus_2	228813_at	204	0.68	0.000370	**-1.57**	0.21 [0.09 - 0.49]

HDAC5

CLASS	DATASET	P.	CANCER TYPE	SUBTYPE	COHORT	AUTHOR	ARRAY TYPE	PROBE ID	N	CUTPOINT	COX P-VALUE	ln(HR)	HR [95% CI low - upp]
IIA	GSE1379	↓	Breast cancer		MGH (1987-2000)	Ma	Arcturus 22k	12814	60	0.87	0.034188	**0.72**	2.05 [1.06 - 3.99]
	GSE4271-GPL97	↑	Brain cancer	Astrocytoma	MDA	Phillips	HG-U133B	229408_at	77	0.31	0.022663	**-0.39**	0.67 [0.48 - 0.95]
	GSE4412-GPL97		Brain cancer	Glioma	UCLA (1996-2003)	Freije	HG-U133B	229408_at	74	0.20	0.029132	**-0.55**	0.58 [0.36 - 0.95]
	GSE17260		Ovarian cancer		Niigata (1997-2008)	Yoshihara	G4112A	A_23_P26922	110	0.11	0.027911	**-0.55**	0.58 [0.35 - 0.94]
	GSE13213		Lung cancer	Adenocarcinoma	Nagoya (1995-1999, 2002-2004)	Tomida	G4112F	A_24_P125283	117	0.39	0.004492	**-0.60**	0.55 [0.36 - 0.83]
	GSE4271-GPL96		Brain cancer	Astrocytoma	MDA	Phillips	HG-U133A	202455_at	77	0.69	0.000468	**-0.71**	0.49 [0.33 - 0.73]
	GSE11121		Breast cancer		Mainz (1988-1998)	Schmidt	HG-U133A	202455_at	200	0.39	0.019412	**-0.91**	0.40 [0.19 - 0.86]
	GSE19615		Breast cancer		DF/HCC	Li	HG-U133_Plus_2	229408_at	115	0.34	0.046196	**-2.04**	0.13 [0.02 - 0.97]
	GSE17537		Colorectal cancer		VMC	Smith	HG-U133_Plus_2	229408_at	55	0.56	0.007193	**-4.97**	0.01 [0.00 - 0.26]
	GSE17537		Colorectal cancer		VMC	Smith	HG-U133_Plus_2	229408_at	55	0.75	0.005719	**-5.39**	0.00 [0.00 - 0.21]
	GSE17537		Colorectal cancer		VMC	Smith	HG-U133_Plus_2	229408_at	49	0.29	0.007959	**-7.03**	0.00 [0.00 - 0.16]

HDAC7

CLASS	DATASET	P.	CANCER TYPE	SUBTYPE	COHORT	AUTHOR	ARRAY TYPE	PROBE ID	N	CUTPOINT	COX P-VALUE	ln(HR)	HR [95% CI low - upp]
IIA	E-DKFZ-1	↓	Renal cell carcinoma		RZPD	Sueltmann	A-RZPD-20	rzpd.de:huber1:Reporter:IMAGE:344782	59	0.78	0.008020	2.89	17.93 [2.12 - 151.47]
	GSE17260	↑	Ovarian cancer		Niigata (1997-2008)	Yoshihara	G4112A	A_23_P2582	110	0.17	0.022718	-0.65	0.52 [0.30 - 0.91]
	GSE13213		Lung cancer	Adenocarcinoma	Nagoya (1995-1999, 2002-2004)	Tomida	G4112F	A_23_P2582	117	0.10	0.019211	-0.66	0.52 [0.30 - 0.90]
	GSE13213		Lung cancer	Adenocarcinoma	Nagoya (1995-1999, 2002-2004)	Tomida	G4112F	A_24_P647146	117	0.21	0.019235	-0.87	0.42 [0.20 - 0.87]

HDAC9

CLASS	DATASET	P.	CANCER TYPE	SUBTYPE	COHORT	AUTHOR	ARRAY TYPE	PROBE ID	N	CUTPOINT	COX P-VALUE	ln(HR)	HR [95% CI low - upp]
IIA	GSE8894	↓	Lung cancer	NSCLC	Seoul (1995-2005)	Lee	HG-U133_Plus_2	234393_at	138	0.90	0.000687	2.08	8.00 [2.41 - 26.59]
	GSE17537		Colorectal cancer		VMC	Smith	HG-U133_Plus_2	205659_at	49	0.90	0.004950	1.42	4.12 [1.53 - 11.04]
	GSE17537		Colorectal cancer		VMC	Smith	HG-U133_Plus_2	205659_at	55	0.89	0.009739	1.04	2.83 [1.29 - 6.24]
	GSE8894		Lung cancer	NSCLC	Seoul (1995-2005)	Lee	HG-U133_Plus_2	1552760_at	138	0.75	0.015691	0.91	2.49 [1.19 - 5.23]
	GSE3141		Lung cancer	NSCLC	Duke	Bild	HG-U133_Plus_2	1552758_at	111	0.74	0.005105	0.38	1.46 [1.12 - 1.90]
	GSE3143		Breast cancer		Duke	Bild	HG-U95A	37483_at	158	0.78	0.016546	0.34	1.41 [1.06 - 1.86]
	GSE16560		Prostate cancer		Sweden (1977-1999)	Sboner	6K DASL	DAP3_3881	281	0.84	0.010652	0.17	1.19 [1.04 - 1.36]
	GSE7390	↑	Breast cancer		Uppsala, Oxford, Stockholm, IGR, GUYT, CRH (1980-1998)	Desmedt	HG-U133A	205659_at	198	0.13	0.019324	-0.34	0.71 [0.54 - 0.95]
	GSE31210		Lung cancer	Adenocarcinoma	NCCRI	Okayama	HG-U133_Plus_2	234393_at	204	0.25	0.002662	-0.53	0.59 [0.42 - 0.83]
	GSE2837		Head and neck cancer	Squamous cell carcinoma	VUMC, VAMC, UTMDACC (1992-2005)	Chung	U133_X3P	Hs2.116753.2.S1_3p_at	28	0.29	0.032335	-0.55	0.57 [0.35 - 0.95]

	GSE1456-GPL96		Breast cancer		Stockholm (1994-1996)	Pawitan	HG-U133A	205659_at	159	0.37	0.035168	**-0.57**	0.56 [0.33 - 0.96]
	GSE1456-GPL96		Breast cancer		Stockholm (1994-1996)	Pawitan	HG-U133A	205659_at	159	0.16	0.009418	**-0.69**	0.50 [0.30 - 0.84]
	GSE1456-GPL96		Breast cancer		Stockholm (1994-1996)	Pawitan	HG-U133A	205659_at	159	0.16	0.021776	**-0.71**	0.49 [0.27 - 0.90]
	GSE17536		Colorectal cancer		MCC	Smith	HG-U133_Plus_2	1552758_at	177	0.11	0.016646	**-2.40**	0.09 [0.01 - 0.65]
	GSE9195		Breast cancer		GUYT2	Loi	HG-U133_Plus_2	234393_at	77	0.44	0.044588	**-4.21**	0.01 [0.00 - 0.90]

HDAC10

CLASS	DATASET	P.	CANCER TYPE	SUBTYPE	COHORT	AUTHOR	ARRAY TYPE	PROBE ID	N	CUTPOINT	COX P-VALUE	ln(HR)	HR [95% CI low - upp]
IIB	GSE9195	↓	Breast cancer		GUYT2	Loi	HG-U133_Plus_2	232870_at	77	0.75	0.036924	**2.70**	14.82 [1.18 - 186.57]
	GSE6532-GPL570		Breast cancer		GUYT	Loi	HG-U133_Plus_2	232870_at	87	0.67	0.014400	**2.21**	9.07 [1.55 - 53.04]
	GSE6532-GPL570		Breast cancer		GUYT	Loi	HG-U133_Plus_2	232870_at	87	0.67	0.014400	**2.21**	9.07 [1.55 - 53.04]
	GSE1456-GPL97		Breast cancer		Stockholm (1994-1996)	Pawitan	HG-U133B	232870_at	159	0.42	0.019625	**0.62**	1.87 [1.11 - 3.15]
	GSE13213	↑	Lung cancer	Adenocarcinoma	Nagoya (1995-1999, 2002-2004)	Tomida	G4112F	A_23_P368740	117	0.11	0.032621	**-0.47**	0.62 [0.40 - 0.96]
	GSE1379		Breast cancer		MGH (1987-2000)	Ma	Arcturus 22k	20695	60	0.23	0.003465	**-3.84**	0.02 [0.00 - 0.28]

HDAC6

CLASS	DATASET	P.	CANCER TYPE	SUBTYPE	COHORT	AUTHOR	ARRAY TYPE	PROBE ID	N	CUTPOINT	COX P-VALUE	ln(HR)	HR [95% CI low - upp]
IIB	E-TABM-158	↓	Breast cancer		UCSF, CPMC (1989-1997)	Chin	HG-U133A	206846_s_at	117	0.56	0.003794	**1.11**	3.03 [1.43 - 6.42]
	E-TABM-158		Breast cancer		UCSF, CPMC (1989-1997)	Chin	HG-U133A	206846_s_at	117	0.56	0.003794	**1.11**	3.03 [1.43 - 6.42]
	GSE13507		Bladder cancer	Transitional cell carcinoma	CNUH	Kim	Human-6 v2	ILMN_1798546	165	0.50	0.039220	**0.60**	1.82 [1.03 - 3.23]
	GSE13507		Bladder cancer		CNUH	Kim	Human-6 v2	ILMN_1798546	165	0.27	0.039062	**0.40**	1.50 [1.02 - 2.20]

	GSE12276		Breast cancer		EMC	Bos	HG-U133_Plus_2	211722_s_at	204	0.47	0.010203	0.25	1.28 [1.06 - 1.55]
	GSE31210		Lung cancer	Adenocarcinoma	NCCRI	Okayama	HG-U133_Plus_2	216224_s_at	204	0.22	0.008007	-0.47	0.62 [0.44 - 0.88]
	GSE9893		Breast cancer		Montpellier, Bordeaux, Turin (1989-2001)	Chanrion	MLRG Human 21K V12.0	7051	155	0.34	0.000130	-0.50	0.61 [0.47 - 0.78]
	GSE2034		Breast cancer		Rotterdam (1980-1995)	Wang	HG-U133A	206846_s_at	286	0.63	0.034782	-0.74	0.48 [0.24 - 0.95]
	jacob-00182-MSK		Lung cancer	Adenocarcinoma	MSK	Shedden	HG-U133A	216224_s_at	104	0.41	0.010084	-1.65	0.19 [0.05 - 0.68]
	GSE17537	↑	Colorectal cancer		VMC	Smith	HG-U133_Plus_2	216224_s_at	55	0.58	0.001299	-3.37	0.03 [0.00 - 0.27]
	GSE17537		Colorectal cancer		VMC	Smith	HG-U133_Plus_2	216224_s_at	49	0.29	0.003833	-3.71	0.02 [0.00 - 0.30]
	GSE17537		Colorectal cancer		VMC	Smith	HG-U133_Plus_2	206846_s_at	49	0.47	0.017342	-3.82	0.02 [0.00 - 0.51]
	GSE17537		Colorectal cancer		VMC	Smith	HG-U133_Plus_2	216224_s_at	55	0.29	0.003821	-3.84	0.02 [0.00 - 0.29]
	GSE17537		Colorectal cancer		VMC	Smith	HG-U133_Plus_2	206846_s_at	55	0.44	0.006084	-3.90	0.02 [0.00 - 0.33]
	GSE8894		Lung cancer	NSCLC	Seoul (1995-2005)	Lee	HG-U133_Plus_2	211722_s_at	138	0.88	0.018497	-3.98	0.02 [0.00 - 0.51]

HDAC11

CLASS	DATASET	P.	CANCER TYPE	SUBTYPE	COHORT	AUTHOR	ARRAY TYPE	PROBE ID	N	CUTPOINT	COX P-VALUE	ln(HR)	HR [95% CI low - upp]
	GSE16581		Brain cancer	Meningioma	UCLA	Lee	HG-U133_Plus_2	219847_at	67	0.54	0.041798	4.07	58.28 [1.16 - 2921.27]
	GSE11595		Esophagus cancer	Adenocarcinoma	Sutton	Giddings	CRUKDMF_22K_v1.0.0	281901	34	0.53	0.030576	2.22	9.18 [1.23 - 68.45]
	GSE6532-GPL570	↓	Breast cancer		GUYT	Loi	HG-U133_Plus_2	219847_at	87	0.64	0.000725	1.33	3.77 [1.75 - 8.15]
IV	GSE6532-GPL570		Breast cancer		GUYT	Loi	HG-U133_Plus_2	219847_at	87	0.64	0.000725	1.33	3.77 [1.75 - 8.15]
	DUKE-OC		Ovarian cancer		Duke	Bild	HG-U133A	219847_at	133	0.61	0.021300	1.15	3.17 [1.19 - 8.45]
	GSE12276		Breast cancer		EMC	Bos	HG-U133_Plus_2	219847_at	204	0.32	0.006443	-0.19	0.83 [0.72 - 0.95]
	GSE7390	↑	Breast cancer		Uppsala, Oxford, Stockholm, IGR, GUYT, CRH (1980-1998)	Desmedt	HG-U133A	219847_at	198	0.17	0.046773	-0.19	0.82 [0.68 - 1.00]

SIRT1

CLASS	DATASET	P.	CANCER TYPE	SUBTYPE	COHORT	AUTHOR	ARRAY TYPE	PROBE ID	N	CUTPOINT	COX P-VALUE	ln(HR)	HR [95% CI low - upp]
III	GSE17536	↓	Colorectal cancer		MCC	Smith	HG-U133_Plus_2	218878_s_at	177	0.90	0.042038	**0.80**	2.22 [1.03 - 4.81]
	GSE17536		Colorectal cancer		MCC	Smith	HG-U133_Plus_2	218878_s_at	177	0.13	0.028564	**0.75**	2.11 [1.08 - 4.13]
	GSE26712		Ovarian cancer		MSKCC (1990-2003)	Bonome	HG-U133_Plus_2	218878_s_at	185	0.78	0.000173	**0.68**	1.98 [1.39 - 2.83]
	GSE26712		Ovarian cancer		MSKCC (1990-2003)	Bonome	HG-U133_Plus_2	218878_s_at	185	0.78	0.001491	**0.54**	1.71 [1.23 - 2.39]
	GSE9893		Breast cancer		Montpellier, Bordeaux, Turin (1989-2001)	Chanrion	MLRG Human 21K V12.0	20060	155	0.83	0.002970	**0.26**	1.30 [1.09 - 1.54]
	DUKE-OC	↑	Ovarian cancer		Duke	Bild	HG-U133A	218878_s_at	133	0.59	0.005605	**-0.41**	0.66 [0.50 - 0.89]
	GSE4271-GPL96		Brain cancer	Astrocytoma	MDA	Phillips	HG-U133A	218878_s_at	77	0.53	0.029271	**-0.70**	0.50 [0.27 - 0.93]
	GSE4412-GPL96		Brain cancer	Glioma	UCLA (1996-2003)	Freije	HG-U133A	218878_s_at	74	0.73	0.000420	**-1.20**	0.30 [0.15 - 0.59]
	GSE1456-GPL96		Breast cancer		Stockholm (1994-1996)	Pawitan	HG-U133A	218878_s_at	159	0.28	0.000514	**-1.85**	0.16 [0.06 - 0.45]
	GSE1456-GPL96		Breast cancer		Stockholm (1994-1996)	Pawitan	HG-U133A	218878_s_at	159	0.28	0.000008	**-2.32**	0.10 [0.04 - 0.27]
	GSE31210		Lung cancer	Adenocarcinoma	NCCRI	Okayama	HG-U133_Plus_2	218878_s_at	204	0.37	0.001453	**-2.56**	0.08 [0.02 - 0.37]
	GSE31210		Lung cancer	Adenocarcinoma	NCCRI	Okayama	HG-U133_Plus_2	218878_s_at	204	0.42	0.000011	**-2.59**	0.08 [0.02 - 0.24]
	GSE1456-GPL96		Breast cancer		Stockholm (1994-1996)	Pawitan	HG-U133A	218878_s_at	159	0.28	0.000006	**-2.87**	0.06 [0.02 - 0.20]

SIRT2

CLASS	DATASET	P.	CANCER TYPE	SUBTYPE	COHORT	AUTHOR	ARRAY TYPE	PROBE ID	N	CUTPOINT	COX P-VALUE	ln(HR)	HR [95% CI low - upp]
III	GSE13507	↓	Bladder cancer	Transitional cell carcinoma	CNUH	Kim	Human-6 v2	ILMN_1723494	165	0.48	0.035885	**0.90**	2.45 [1.06 - 5.67]
	GSE1379		Breast cancer		MGH (1987-2000)	Ma	Arcturus 22k	19342	60	0.88	0.003736	**0.86**	2.36 [1.32 - 4.20]

	GSE9891		Ovarian cancer		AOCS, RBH, WH, NKI-AVL (1992-2006)	Tothill	HG-U133_Plus_2	1558331_at	278	0.84	0.012910	0.42	1.52 [1.09 - 2.11]
	DUKE-OC		Ovarian cancer		Duke	Bild	HG-U133A	220605_s_at	133	0.87	0.020304	0.36	1.43 [1.06 - 1.94]
	GSE31210		Lung cancer	Adenocarcinoma	NCCRI	Okayama	HG-U133_Plus_2	1558331_at	204	0.58	0.020039	-0.42	0.66 [0.46 - 0.94]
	GSE31210		Lung cancer	Adenocarcinoma	NCCRI	Okayama	HG-U133_Plus_2	1558331_at	204	0.58	0.009042	-0.55	0.58 [0.38 - 0.87]
	GSE4271-GPL96	↑	Brain cancer	Astrocytoma	MDA	Phillips	HG-U133A	220605_s_at	77	0.17	0.001496	-1.18	0.31 [0.15 - 0.64]
	GSE17537		Colorectal cancer		VMC	Smith	HG-U133_Plus_2	1558331_at	49	0.16	0.020039	-3.51	0.03 [0.00 - 0.58]
	GSE17537		Colorectal cancer		VMC	Smith	HG-U133_Plus_2	1558331_at	55	0.11	0.011806	-3.52	0.03 [0.00 - 0.46]

SIRT3

CLASS	DATASET	P.	CANCER TYPE	SUBTYPE	COHORT	AUTHOR	ARRAY TYPE	PROBE ID	N	CUTPOINT	COX P-VALUE	ln(HR)	HR [95% CI low - upp]
	GSE19234		Skin cancer	Melanoma	NYU	Bogunovic	HG-U133_Plus_2	221913_at	38	0.55	0.020717	1.68	5.38 [1.29 - 22.38]
	GSE19234		Skin cancer	Melanoma	NYU	Bogunovic	HG-U133_Plus_2	221562_s_at	38	0.87	0.032540	1.08	2.94 [1.09 - 7.90]
	E-TABM-158	↓	Breast cancer		UCSF, CPMC (1989-1997)	Chin	HG-U133A	221913_at	117	0.83	0.041052	0.82	2.27 [1.03 - 4.99]
	GSE9893		Breast cancer		Montpellier, Bordeaux, Turin (1989-2001)	Chanrion	MLRG Human 21K V12.0	18003	155	0.65	0.000935	0.34	1.40 [1.15 - 1.71]
	GSE12276		Breast cancer		EMC	Bos	HG-U133_Plus_2	221562_s_at	204	0.37	0.039536	-0.27	0.76 [0.59 - 0.99]
III	GSE2034		Breast cancer		Rotterdam (1980-1995)	Wang	HG-U133A	221562_s_at	286	0.13	0.019214	-0.30	0.74 [0.58 - 0.95]
	GSE3494-GPL96		Breast cancer		Uppsala (1987-1989)	Miller	HG-U133A	221562_s_at	236	0.46	0.033368	-0.35	0.71 [0.51 - 0.97]
	GSE12276	↑	Breast cancer		EMC	Bos	HG-U133_Plus_2	221913_at	204	0.22	0.005261	-0.44	0.64 [0.47 - 0.88]
	GSE13213		Lung cancer	Adenocarcinoma	Nagoya (1995-1999, 2002-2004)	Tomida	G4112F	A_23_P139265	117	0.71	0.049923	-0.45	0.64 [0.41 - 1.00]
	GSE2034		Breast cancer		Rotterdam (1980-1995)	Wang	HG-U133A	221913_at	286	0.50	0.010092	-0.49	0.61 [0.42 - 0.89]
	GSE14333		Colorectal cancer		Melbourne	Jorissen	HG-U133_Plus_2	221562_s_at	226	0.12	0.007479	-0.51	0.60 [0.41 - 0.87]

	GSE3494-GPL96		Breast cancer		Uppsala (1987-1989)	Miller	HG-U133A	221913_at	236	0.18	0.046545	**-0.68**	0.51 [0.26 - 0.99]
	GSE12276		Breast cancer		EMC	Bos	HG-U133_Plus_2	49327_at	204	0.41	0.001536	**-0.70**	0.50 [0.32 - 0.77]
	GSE1456-GPL96		Breast cancer		Stockholm (1994-1996)	Pawitan	HG-U133A	221913_at	159	0.11	0.028806	**-0.72**	0.49 [0.25 - 0.93]
	GSE1456-GPL96		Breast cancer		Stockholm (1994-1996)	Pawitan	HG-U133A	221913_at	159	0.21	0.007705	**-0.75**	0.47 [0.27 - 0.82]
	GSE11121		Breast cancer		Mainz (1988-1998)	Schmidt	HG-U133A	221562_s_at	200	0.12	0.008191	**-0.79**	0.45 [0.25 - 0.82]
	GSE31210		Lung cancer	Adenocarcinoma	NCCRI	Okayama	HG-U133_Plus_2	221913_at	204	0.42	0.034104	**-0.81**	0.44 [0.21 - 0.94]
	jacob-00182-UM		Lung cancer	Adenocarcinoma	UM	Shedden	HG-U133A	221562_s_at	178	0.44	0.000322	**-1.07**	0.34 [0.19 - 0.62]
	GSE4412-GPL96		Brain cancer	Glioma	UCLA (1996-2003)	Freije	HG-U133A	221913_at	74	0.76	0.013741	**-1.19**	0.30 [0.12 - 0.78]
	GSE9195		Breast cancer		GUYT2	Loi	HG-U133_Plus_2	221913_at	77	0.39	0.047916	**-1.42**	0.24 [0.06 - 0.99]
	jacob-00182-MSK		Lung cancer	Adenocarcinoma	MSK	Shedden	HG-U133A	221913_at	104	0.27	0.022969	**-1.82**	0.16 [0.03 - 0.78]
	GSE31210		Lung cancer	Adenocarcinoma	NCCRI	Okayama	HG-U133_Plus_2	49327_at	204	0.10	0.028381	**-1.91**	0.15 [0.03 - 0.82]
	GSE31210		Lung cancer	Adenocarcinoma	NCCRI	Okayama	HG-U133_Plus_2	49327_at	204	0.23	0.000254	**-2.35**	0.10 [0.03 - 0.34]
	GSE9195		Breast cancer		GUYT2	Loi	HG-U133_Plus_2	49327_at	77	0.16	0.020687	**-2.81**	0.06 [0.01 - 0.65]

SIRT4

CLASS	DATASET	P.	CANCER TYPE	SUBTYPE	COHORT	AUTHOR	ARRAY TYPE	PROBE ID	N	CUTPOINT	COX P-VALUE	ln(HR)	HR [95% CI low - upp]
III	DUKE-OC	↓	Ovarian cancer		Duke	Bild	HG-U133A	222248_s_at	133	0.85	0.012273	**3.96**	52.41 [2.36 - 1161.86]
	GSE9891		Ovarian cancer		AOCS, RBH, WH, NKI-AVL (1992-2006)	Tothill	HG-U133_Plus_2	222248_s_at	278	0.77	0.002569	**0.56**	1.76 [1.22 - 2.53]
	GSE9891		Ovarian cancer		AOCS, RBH, WH, NKI-AVL (1992-2006)	Tothill	HG-U133_Plus_2	220047_at	278	0.72	0.003043	**0.55**	1.74 [1.21 - 2.50]
	GSE9893	↑	Breast cancer		Montpellier, Bordeaux, Turin (1989-2001)	Chanrion	MLRG Human 21K V12.0	7747	155	0.22	0.001350	**-0.45**	0.64 [0.48 - 0.84]
	GSE1456-GPL96		Breast cancer		Stockholm (1994-1996)	Pawitan	HG-U133A	222248_s_at	159	0.10	0.035967	**-0.59**	0.55 [0.32 - 0.96]

	GSE1456-GPL96		Breast cancer		Stockholm (1994-1996)	Pawitan	HG-U133A	222248_s_at	159	0.10	0.005777	**-0.68**	0.51 [0.31 - 0.82]
	GSE31210		Lung cancer	Adenocarcinoma	NCCRI	Okayama	HG-U133_Plus_2	220047_at	204	0.14	0.002626	**-0.81**	0.44 [0.26 - 0.75]
	GSE31210		Lung cancer	Adenocarcinoma	NCCRI	Okayama	HG-U133_Plus_2	220047_at	204	0.39	0.003900	**-1.02**	0.36 [0.18 - 0.72]
	GSE9195		Breast cancer		GUYT2	Loi	HG-U133_Plus_2	220047_at	77	0.12	0.046315	**-1.32**	0.27 [0.07 - 0.98]

SIRT5

CLASS	DATASET	P.	CANCER TYPE	SUBTYPE	COHORT	AUTHOR	ARRAY TYPE	PROBE ID	N	CUTPOINT	COX P-VALUE	ln(HR)	HR [95% CI low - upp]
III	GSE9195	↓	Breast cancer		GUYT2	Loi	HG-U133_Plus_2	1569938_at	77	0.90	0.001346	**4.56**	95.66 [5.89 - 1554.71]
	GSE9195		Breast cancer		GUYT2	Loi	HG-U133_Plus_2	1569938_at	77	0.90	0.028742	**2.90**	18.15 [1.35 - 243.78]
	GSE17537		Colorectal cancer		VMC	Smith	HG-U133_Plus_2	219185_at	49	0.88	0.002093	**2.48**	11.89 [2.46 - 57.60]
	GSE11121		Breast cancer		Mainz (1988-1998)	Schmidt	HG-U133A	219185_at	200	0.86	0.000637	**1.54**	4.67 [1.93 - 11.30]
	GSE19615		Breast cancer		DF/HCC	Li	HG-U133_Plus_2	221010_s_at	115	0.75	0.029209	**1.49**	4.45 [1.16 - 17.00]
	GSE19234		Skin cancer	Melanoma	NYU	Bogunovic	HG-U133_Plus_2	221010_s_at	38	0.37	0.006120	**1.40**	4.06 [1.49 - 11.04]
	GSE19234		Skin cancer	Melanoma	NYU	Bogunovic	HG-U133_Plus_2	219185_at	38	0.71	0.035125	**1.08**	2.94 [1.08 - 8.04]
	GSE13213		Lung cancer	Adenocarcinoma	Nagoya (1995-1999, 2002-2004)	Tomida	G4112F	A_23_P307955	117	0.68	0.007575	**0.45**	1.57 [1.13 - 2.19]
	GSE26712		Ovarian cancer		MSKCC (1990-2003)	Bonome	HG-U133_Plus_2	219185_at	185	0.74	0.014893	**-0.34**	0.71 [0.54 - 0.94]
	GSE26712		Ovarian cancer		MSKCC (1990-2003)	Bonome	HG-U133_Plus_2	219185_at	185	0.74	0.010352	**-0.40**	0.67 [0.49 - 0.91]
	GSE31210		Lung cancer	Adenocarcinoma	NCCRI	Okayama	HG-U133_Plus_2	1569938_at	204	0.48	0.026736	**-0.41**	0.66 [0.46 - 0.95]
	GSE9893	↑	Breast cancer		Montpellier, Bordeaux, Turin (1989-2001)	Chanrion	MLRG Human 21K V12.0	9296	155	0.26	0.003489	**-0.58**	0.56 [0.38 - 0.83]
	GSE4412-GPL96		Brain cancer	Glioma	UCLA (1996-2003)	Freije	HG-U133A	219185_at	74	0.47	0.022556	**-1.75**	0.17 [0.04 - 0.78]
	GSE16581		Brain cancer	Meningioma	UCLA	Lee	HG-U133_Plus_2	1569938_at	67	0.34	0.017036	**-10.84**	0.00 [0.00 - 0.14]

	GSE8894		Lung cancer	NSCLC	Seoul (1995-2005)	Lee	HG-U133_Plus_2	1569938_at	138	0.75	0.004701	-3.66	0.03 [0.00 - 0.33]

SIRT6

CLASS	DATASET	P.	CANCER TYPE	SUBTYPE	COHORT	AUTHOR	ARRAY TYPE	PROBE ID	N	CUTPOINT	COX P-VALUE	ln(HR)	HR [95% CI low - upp]
	GSE1456-GPL97		Breast cancer		Stockholm (1994-1996)	Pawitan	HG-U133B	233179_x_at	159	0.75	0.001891	2.20	9.06 [2.26 - 36.36]
	GSE1456-GPL97		Breast cancer		Stockholm (1994-1996)	Pawitan	HG-U133B	233179_x_at	159	0.73	0.000632	2.11	8.29 [2.46 - 27.87]
	GSE3494-GPL96		Breast cancer		Uppsala (1987-1989)	Miller	HG-U133A	219613_s_at	236	0.52	0.001793	1.90	6.70 [2.03 - 22.13]
	GSE4922-GPL96		Breast cancer		Uppsala (1987-1989)	Ivshina	HG-U133A	219613_s_at	249	0.51	0.000276	1.76	5.81 [2.25 - 15.00]
	GSE1456-GPL97		Breast cancer		Stockholm (1994-1996)	Pawitan	HG-U133B	233179_x_at	159	0.75	0.017005	1.47	4.34 [1.30 - 14.52]
	GSE17537	↓	Colorectal cancer		VMC	Smith	HG-U133_Plus_2	234361_at	55	0.89	0.034295	1.47	4.35 [1.11 - 16.97]
	GSE31210		Lung cancer	Adenocarcinoma	NCCRI	Okayama	HG-U133_Plus_2	219613_s_at	204	0.66	0.038428	1.36	3.88 [1.07 - 14.00]
III	GSE26712		Ovarian cancer		MSKCC (1990-2003)	Bonome	HG-U133_Plus_2	219613_s_at	185	0.19	0.010806	0.92	2.51 [1.24 - 5.08]
	GSE26712		Ovarian cancer		MSKCC (1990-2003)	Bonome	HG-U133_Plus_2	219613_s_at	185	0.89	0.025787	0.87	2.39 [1.11 - 5.14]
	GSE4412-GPL97		Brain cancer	Glioma	UCLA (1996-2003)	Freije	HG-U133B	234361_at	74	0.85	0.047730	0.47	1.59 [1.00 - 2.53]
	GSE4271-GPL97		Brain cancer	Astrocytoma	MDA	Phillips	HG-U133B	234361_at	77	0.79	0.006239	-0.46	0.63 [0.45 - 0.88]
	GSE2034		Breast cancer		Rotterdam (1980-1995)	Wang	HG-U133A	219613_s_at	286	0.26	0.016588	-0.57	0.57 [0.35 - 0.90]
	GSE3141	↑	Lung cancer	NSCLC	Duke	Bild	HG-U133_Plus_2	219613_s_at	111	0.28	0.024389	-1.16	0.31 [0.11 - 0.86]
	GSE17537		Colorectal cancer		VMC	Smith	HG-U133_Plus_2	233179_x_at	55	0.29	0.014310	-2.27	0.10 [0.02 - 0.64]
	GSE17537		Colorectal cancer		VMC	Smith	HG-U133_Plus_2	233179_x_at	55	0.13	0.003410	-3.15	0.04 [0.01 - 0.35]

SIRT7

CLASS	DATASET	P.	CANCER TYPE	SUBTYPE	COHORT	AUTHOR	ARRAY TYPE	PROBE ID	N	CUTPOINT	COX P-VALUE	ln(HR)	HR [95% CI low - upp]

	GSE7378		Breast cancer		UCSF	Zhou	U133AAofAv2	218797_s_at	54	0.83	0.011161	**1.86**	6.41 [1.53 - 26.90]
	GSE19234		Skin cancer	Melanoma	NYU	Bogunovic	HG-U133_Plus_2	218797_s_at	38	0.32	0.039076	**1.06**	2.90 [1.05 - 7.98]
	jacob-00182-HLM		Lung cancer	Adenocarcinoma	HLM	Shedden	HG-U133A	218797_s_at	79	0.63	0.035436	**1.04**	2.82 [1.07 - 7.41]
	GSE4271-GPL96	↓	Brain cancer	Astrocytoma	MDA	Phillips	HG-U133A	218797_s_at	77	0.65	0.045592	**0.89**	2.43 [1.02 - 5.82]
III	GSE31210		Lung cancer	Adenocarcinoma	NCCRI	Okayama	HG-U133_Plus_2	218797_s_at	204	0.52	0.024068	**0.83**	2.28 [1.11 - 4.68]
	GSE9893		Breast cancer		Montpellier, Bordeaux, Turin (1989-2001)	Chanrion	MLRG Human 21K V12.0	20594	155	0.80	0.005883	**0.38**	1.46 [1.12 - 1.92]
	GSE26712		Ovarian cancer		MSKCC (1990-2003)	Bonome	HG-U133_Plus_2	218797_s_at	185	0.85	0.023843	**-0.39**	0.68 [0.48 - 0.95]
	GSE26712	↑	Ovarian cancer		MSKCC (1990-2003)	Bonome	HG-U133_Plus_2	218797_s_at	185	0.83	0.033287	**-0.41**	0.67 [0.46 - 0.97]
	GSE17537		Colorectal cancer		VMC	Smith	HG-U133_Plus_2	218797_s_at	55	0.22	0.043732	**-1.34**	0.26 [0.07 - 0.96]

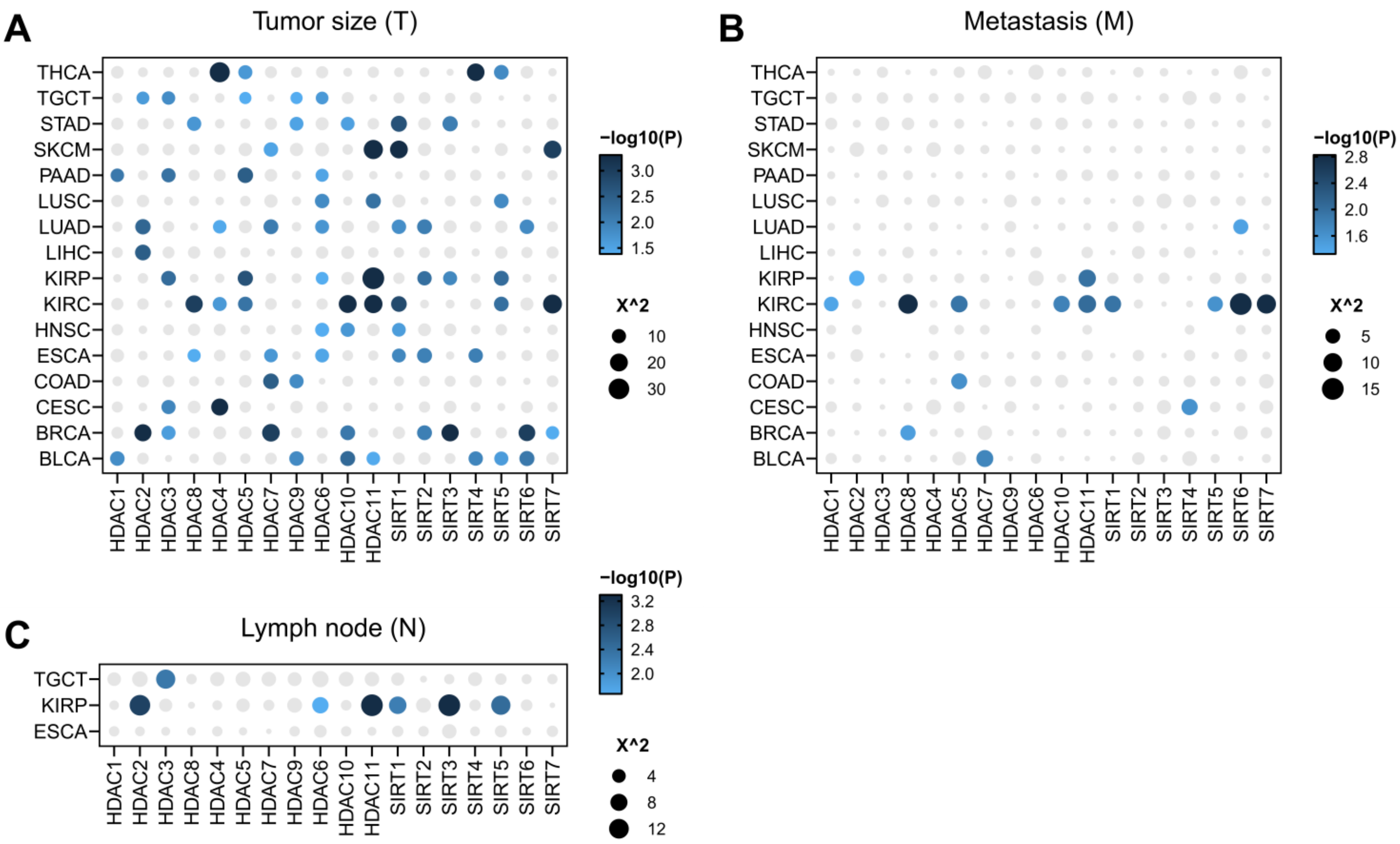

Figure S4. Chi-squared analysis of the correlation between HDAC family members' high (Q3, 75th percentile) relative to low (Q1, 25th percentile) expression levels and clinicopathological features of TNM classification across TCGA studies. (A) Tumor size. (B) Metastasis. (C) Lymph nodes. Dots size and color gradient represent X^2 values and p-value<0.05, respectively. Continuity correction applied. COAD stands here as a double abbreviation for COADREAD.

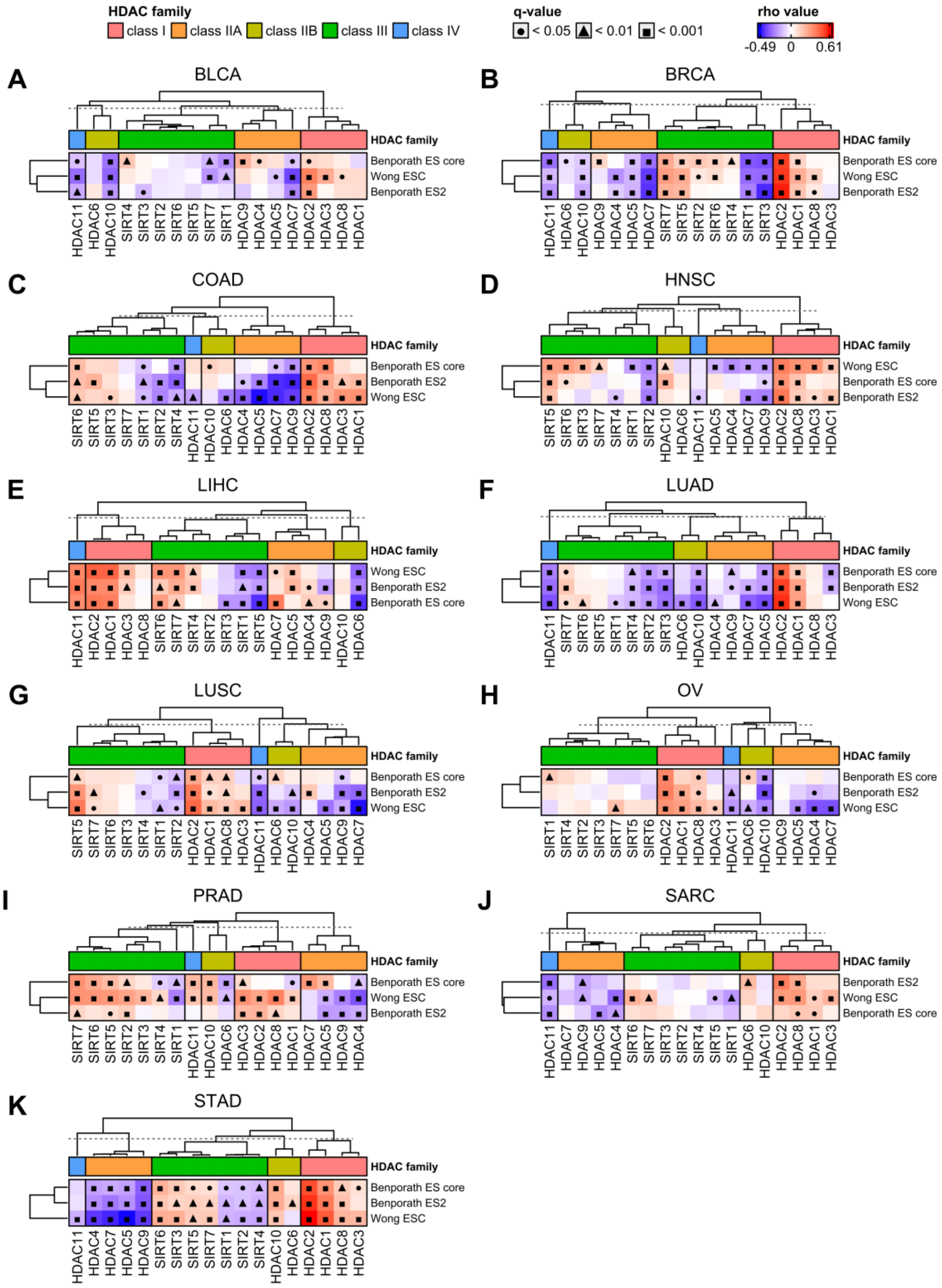

Figure S5. Correlations between expression of HDAC family genes and adequate GSVA stemness scores (Wong ESC, Benporath ES2, and Benporath ES core) in selected TCGA studies: (A) bladder urothelial carcinoma, (B) breast invasive carcinoma, (C) colon adenocarcinoma, (D) head and neck squamous cell carcinoma, (E) liver hepatocellular carcinoma, (F) lung adenocarcinoma, (G) lung squamous cell carcinoma, (H) ovarian serous cystadenocarcinoma, (I) prostate adenocarcinoma, (J) sarcoma, and (K) stomach adenocarcinoma. Spearman's test with an asymptotic t-test for p-values. Benjamin-Hochberg for multiple testing corrections.

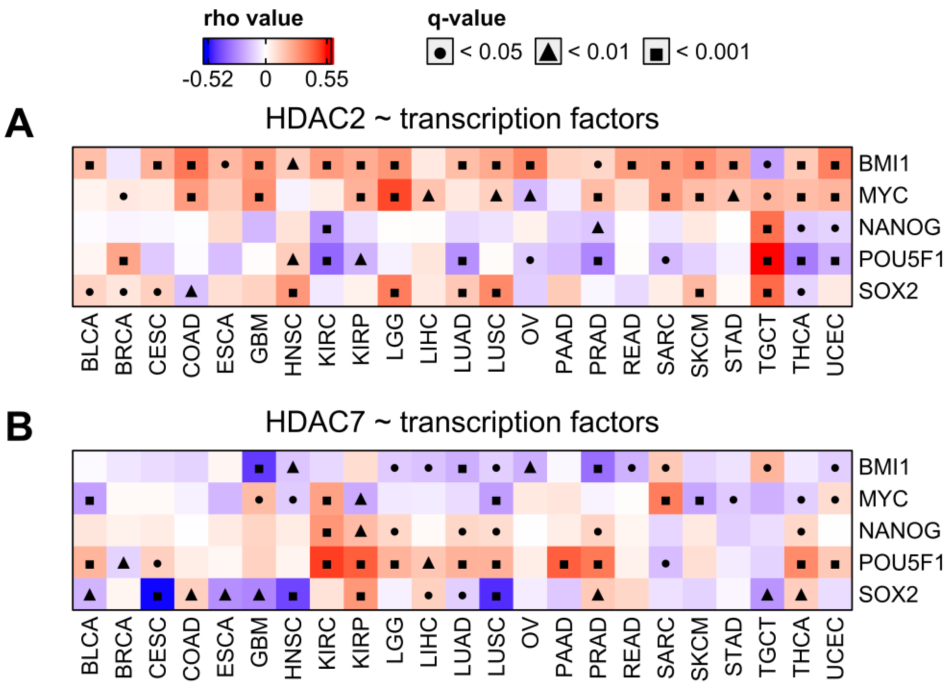

Figure S6. Correlations of pluripotency transcription factors (POU5F1, SOX2, NANOG, MYC, BMI1) with HDAC2 (A) and HDAC7 (B) gene expression in GDC PANCAN dataset (including only TCGA studies of solid tumors with more than 100 RNA-seqV2 available samples). Spearman's test with asymptotic t-test for p-values. Benjamin-Hochberg for multiple testing corrections.

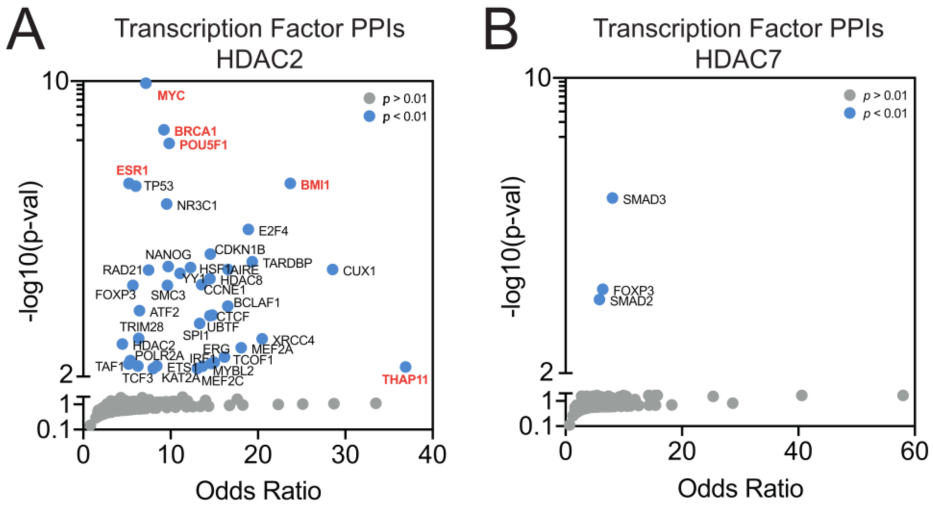

Figure S7. Enrichment analysis to detect potential targets for known transcription factors associated with (A) the HDAC2-related and (B) the HDAC7-related gene expression profiles. The top 100 most relevant genes were identified with the ARCHS4 RNA-seq gene-gene co-expression matrix for each gene (Enrichr tool, Transcription Factor PPIs module). Genes marked red are known pluripotency markers.

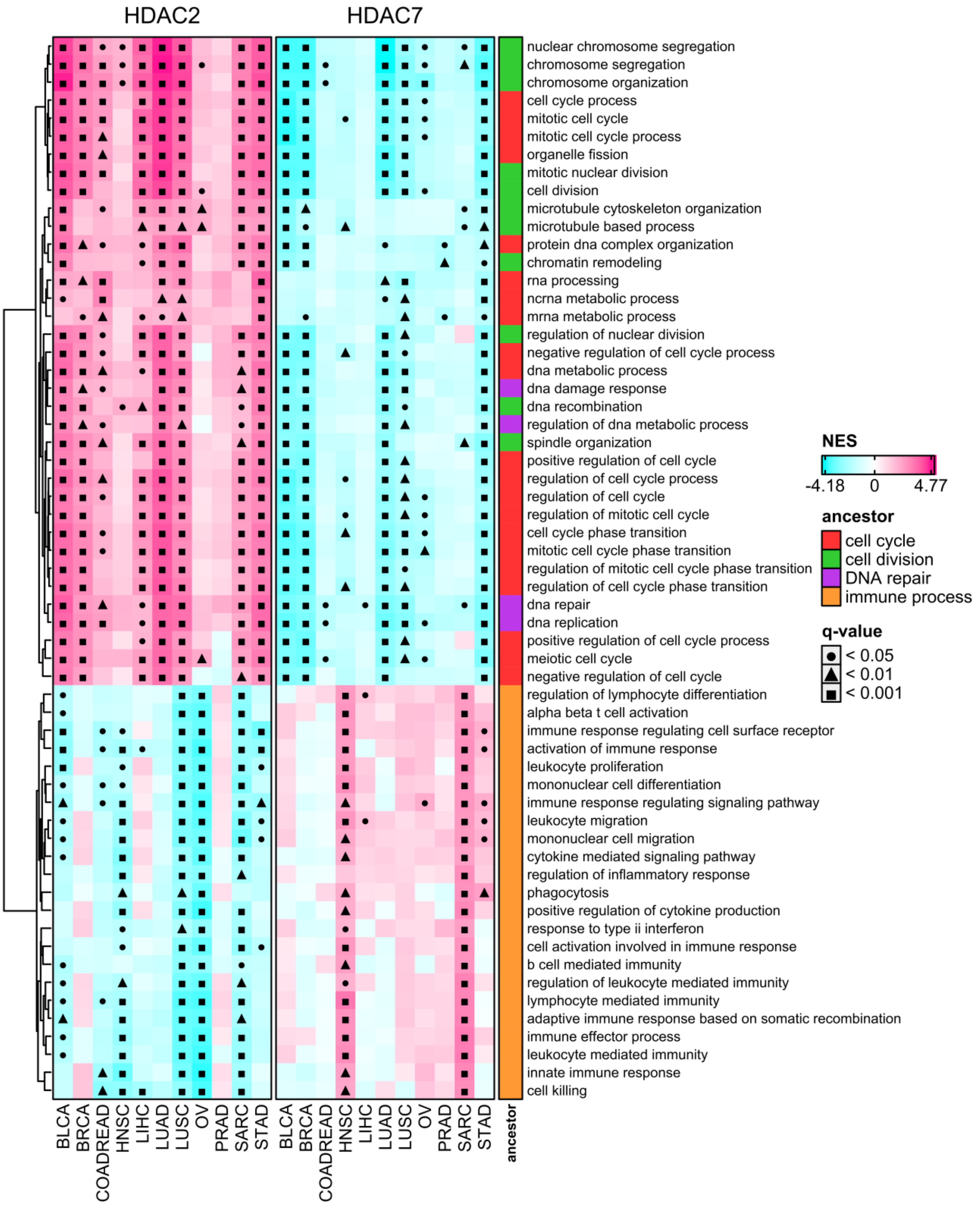

Figure S8. C5:BP Gene Set Enrichment (GSEA) preranked analysis of HDAC2 and HDAC7 gene expression across selected TCGA studies. The high expression group (Q3, 75th percentile) relative to the low (Q1, 25th percentile) expression group. Negative and positive NES values indicate the enrichment of selected gene sets from GO Biological Process ontology collection (CP5) at the bottom or the top of the ranked data sets. Gray cells indicate no available data. Benjamin-Hochberg for multiple testing corrections.

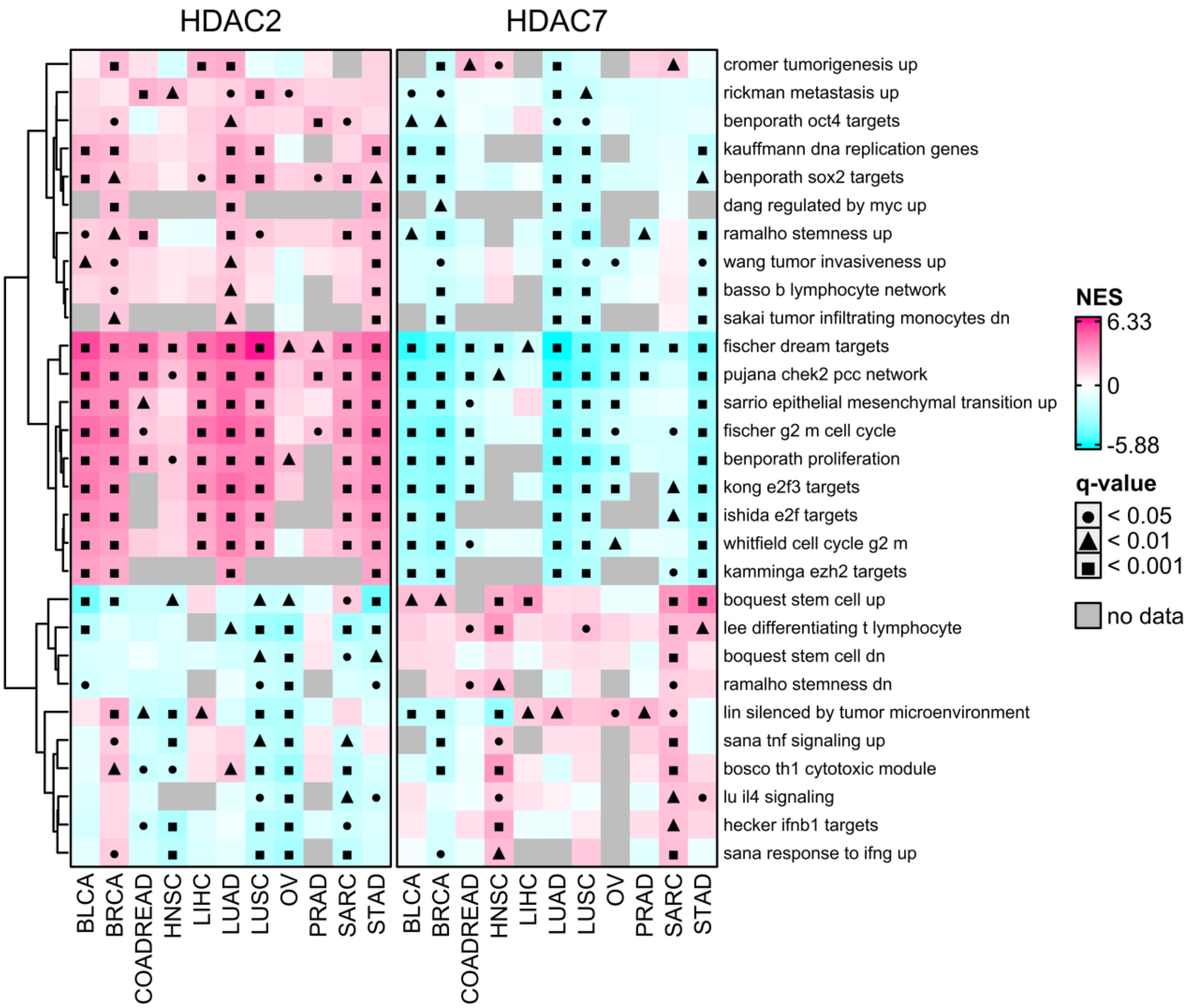

Figure S9. C2:CGP Gene Set Enrichment (GSEA) preranked analysis of HDAC2 and HDAC7 gene expression across selected TCGA studies. The high expression group (Q3, 75th percentile) relative to the low (Q1, 25th percentile) expression group. Negative and positive NES values indicate the enrichment of selected curated gene sets from chemical and genetic perturbations collection (CP2) at the bottom or the top of the ranked data set. Gray cells indicate no available data. Benjamin-Hochberg for multiple testing corrections

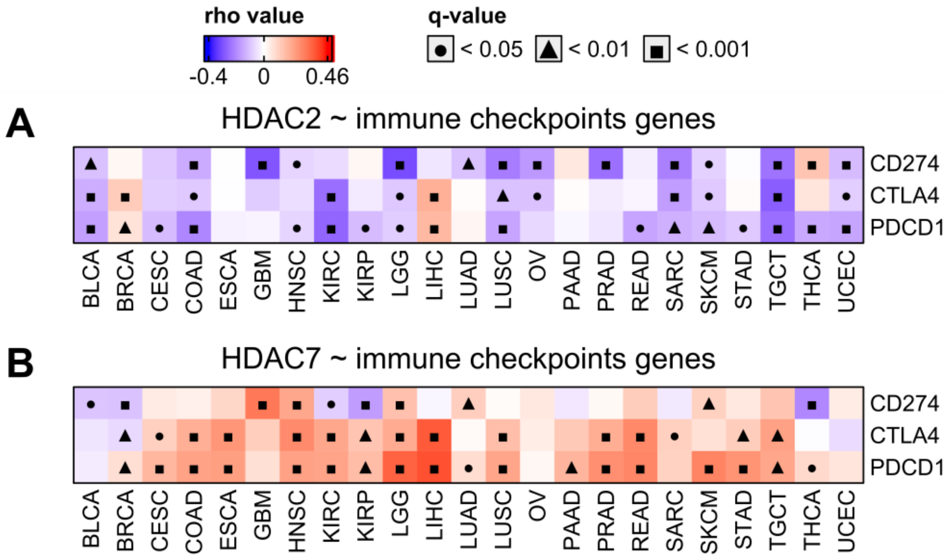

Figure S10. Correlations of immune checkpoint proteins (PD-1, PD-L1, CTLA4) with HDAC2 (A) and HDAC7 (B) gene expression in GDC PANCAN dataset derived from the USCS Xena Browser (including only TCGA studies of solid tumors with more than 100 RNA-seqV2 available samples). Spearman's test with asymptotic t-test for p-values. Benjamin-Hochberg for multiple testing corrections.

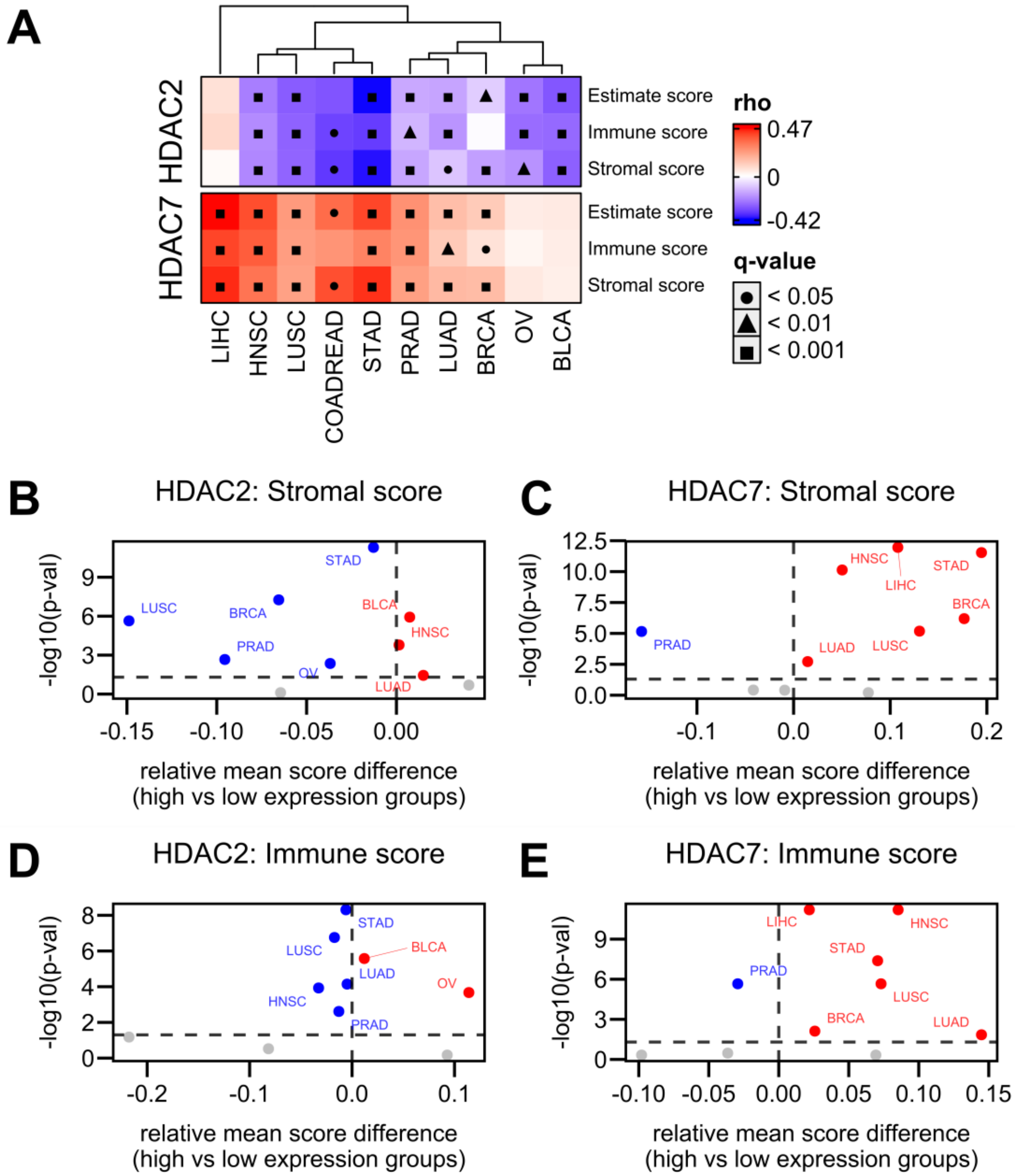

Figure S11. ESTIMATE (Estimation of Stromal and Immune cells in Malignant Tumor tissues using Expression data) analysis. (A) Correlations between HDAC2 and HDAC7 gene expression with ESTIMATE expression-based scores (RNA-seqV2 samples) for selected TCGA studies calculated by the MD Anderson Cancer Center. Score for the tumor purity (Estimate score), the level of stromal cells present in tumor tissues (Stromal score), and the infiltration level of immune cells (Immune score). SARC results were unavailable in this database. Spearman's test with asymptotic t-test for p-values. Benjamin-Hochberg for multiple testing corrections. (B-E) Differences of (B-C) Stromal and (D-E) Immune scores between high (Q3, 75th percentile) and low (Q1, 25th percentile) expression level groups of (B,D) HDAC2 and (C,E) HDAC7 in selected TCGA studies. The X axis represents the difference of mean score (with prior min-max normalization) in high relative to low expression groups. Each color-coded dot represents one study: red if the score is higher in the high expression group compared to the low one, and blue if the score is higher in the low expression group compared to the high one. Mann-Whitney U test / Wilcoxon sum-rank test. Benjamin-Hochberg for multiple testing corrections.

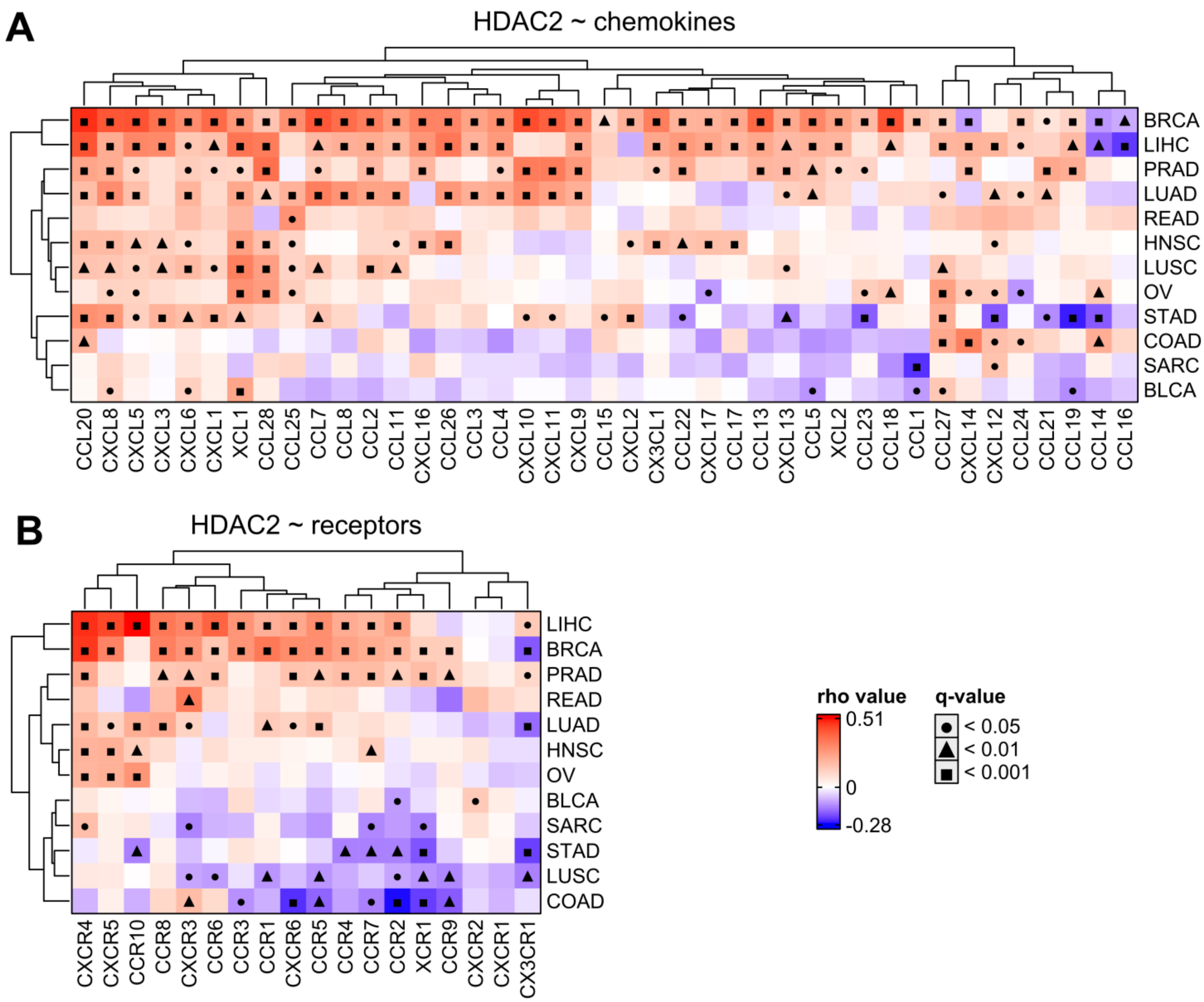

Figure S12. Expression correlations of HDAC2 with (A) chemokines and (B) chemokine receptors across distinct tumor types. Spearman's test with asymptotic t-test for p-values. Benjamin-Hochberg for multiple testing corrections.

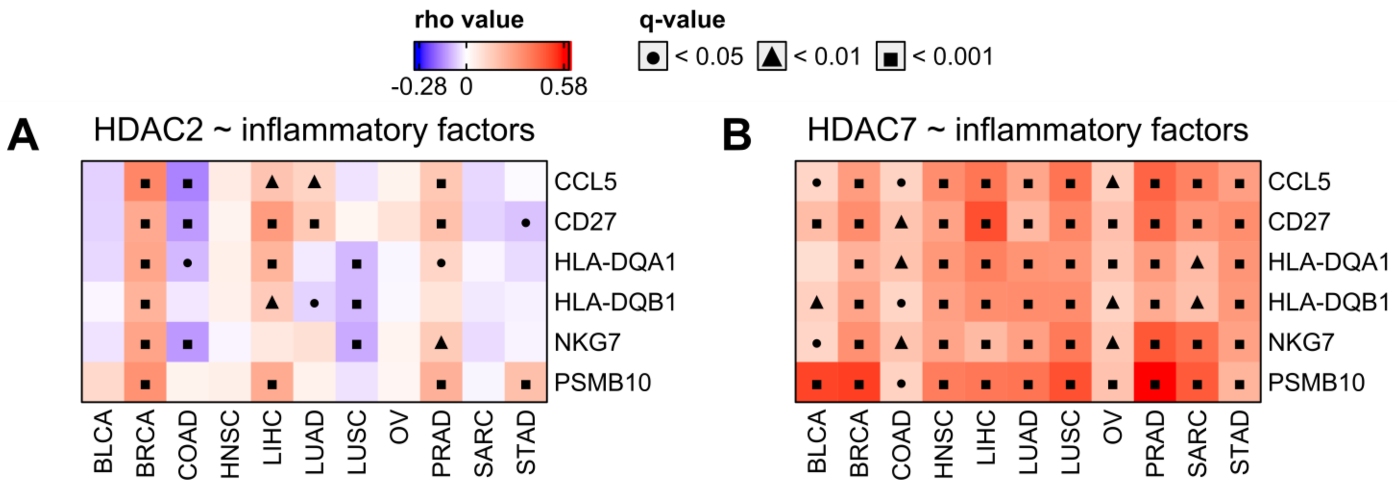

Figure S13. HDAC7 expression is significantly associated with the promotion of inflammatory factors. Correlations of inflammation-related genes (CCL5, CD27, HLA-DQA1, HLA-DQB1, NKG7, PSMB10 with (A) HDAC2 and (B) HDAC7 gene expression in GDC PANCAN dataset derived from the UCSC Xena Browser (including only TCGA studies of solid tumors with more than 100 RNA-seqV2 available samples). Spearman's test with asymptotic t-test for p-values. Benjamin-Hochberg for multiple testing corrections.

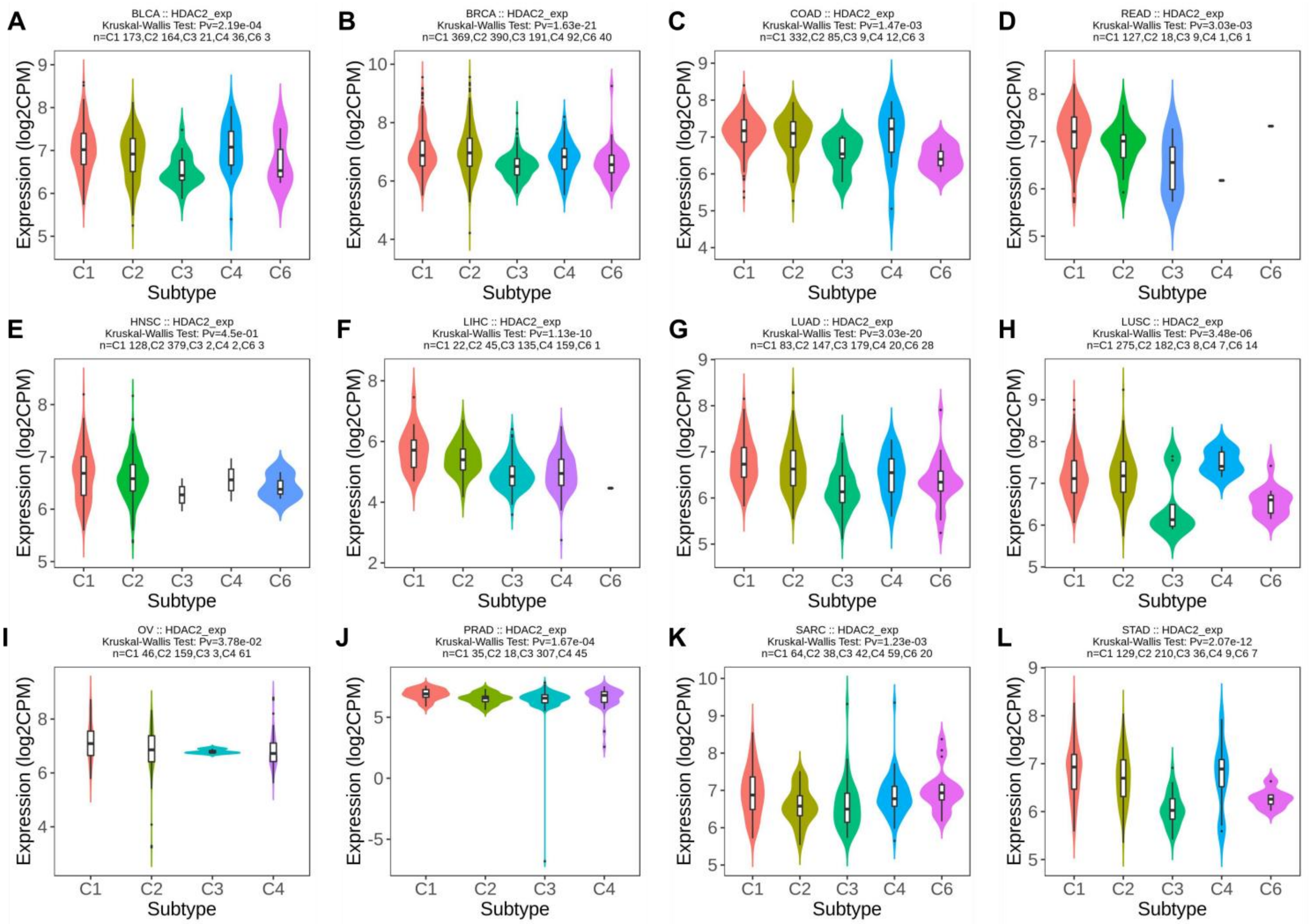

Figure S14. The distribution of HDAC2 gene expression across immune subtypes in selected TCGA studies: (A) bladder urothelial carcinoma, (B) breast invasive carcinoma, (C) colon adenocarcinoma, (D) rectum adenocarcinoma, (E) head and neck squamous cell carcinoma, (F) liver hepatocellular carcinoma, (G) lung adenocarcinoma, (H) lung squamous cell carcinoma, (I) ovarian serous cystadenocarcinoma, (J) prostate adenocarcinoma, (K) sarcoma, and (L) stomach adenocarcinoma. Panels with statistics downloaded directly from the TISIDB database. P-values (Pv) of the Kruskal-Wallis test for the comparison of expression levels across immune subtypes, denoted as follows: (C1) wound healing, (C2) IFN-gamma dominant, (C3) inflammatory, (C4) lymphocyte depleted, (C6) TGF-b dominant.

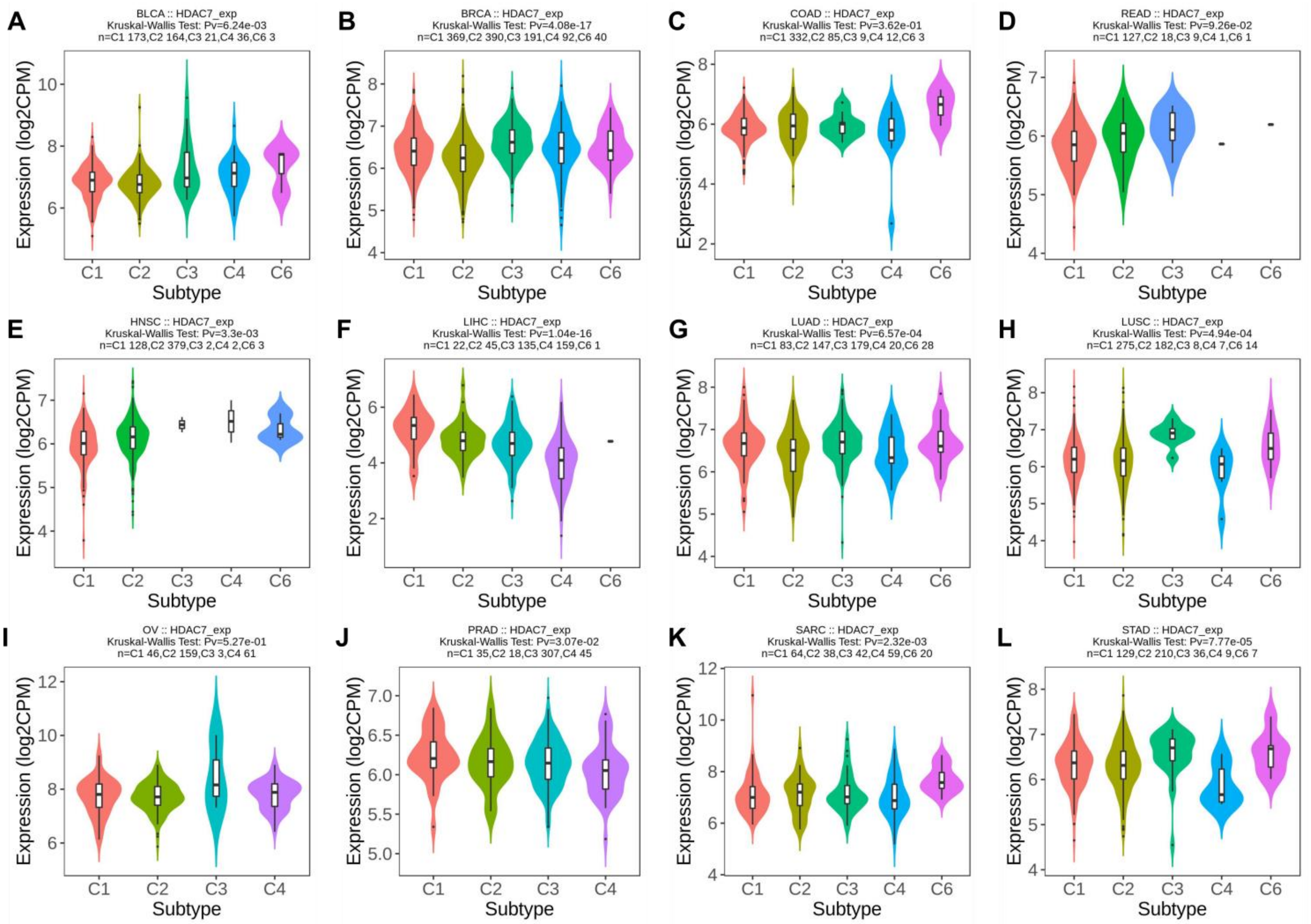

Figure S15. The distribution of HDAC7 gene expression across immune subtypes in selected TCGA studies: (A) bladder urothelial carcinoma, (B) breast invasive carcinoma, (C) colon adenocarcinoma, (D) rectum adenocarcinoma, (E) head and neck squamous cell carcinoma, (F) liver hepatocellular carcinoma, (G) lung adenocarcinoma, (H) lung squamous cell carcinoma, (I) ovarian serous cystadenocarcinoma, (J) prostate adenocarcinoma, (K) sarcoma, and (L) stomach adenocarcinoma. Panels with statistics downloaded directly from the TISIDB database. P-values (Pv) of the Kruskal-Wallis test for the comparison of expression levels across immune subtypes, denoted as follows: (C1) wound healing, (C2) IFN-gamma dominant, (C3) inflammatory, (C4) lymphocyte depleted, (C6) TGF-b dominant.

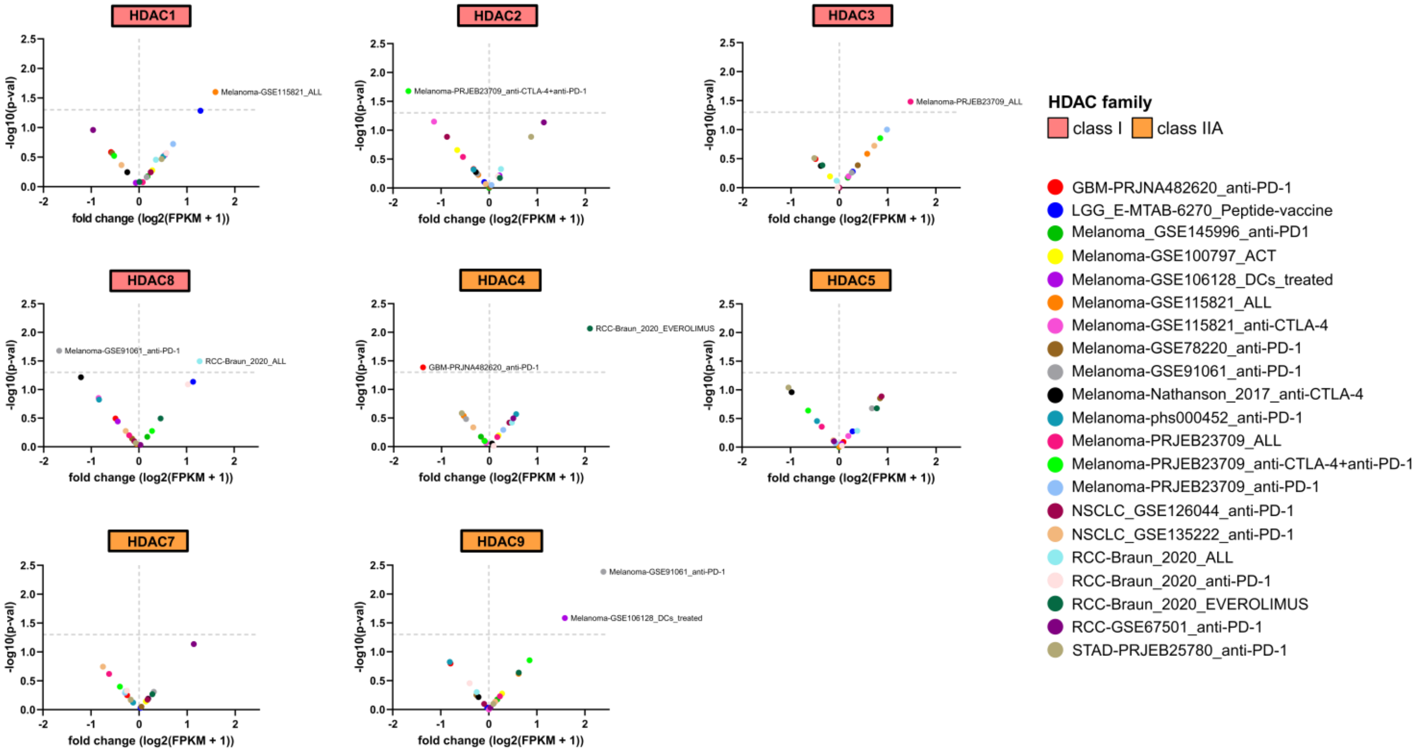

Figure S16. Differential expression of class I and class IIA HDACs between responders and non-responders to immunotherapy in different tumors. Volcano plots present the fold change (log2FC) of HDACs' expression against statistical significance (-log10(p-val)). Specific datasets from the TIGER database are color-coded.
